# Discovery of antimicrobial peptides clostrisin and cellulosin from Clostridium: insights into their structures, co-localized biosynthetic gene clusters, and antibiotic activity

**DOI:** 10.3762/bjoc.20.159

**Published:** 2024-07-30

**Authors:** Moisés Alejandro Alejo Hernandez, Katia Pamela Villavicencio Sánchez, Rosendo Sánchez Morales, Karla Georgina Hernández-Magro Gil, David Silverio Moreno-Gutiérrez, Eddie Guillermo Sanchez-Rueda, Yanet Teresa-Cruz, Brian Choi, Armando Hernández Garcia, Alba Romero-Rodríguez, Oscar Juárez, Siseth Martínez-Caballero, Mario Figueroa, Corina-Diana Ceapă

**Affiliations:** 1 Laboratory of Microbiology, Institute of Chemistry, National Autonomous University of Mexico , Mexico City, 04510, Mexico; 2 Biomolecular Engineering and Bionanotechnology Laboratory, Institute of Chemistry, National Autonomous University of Mexico , Mexico City, 04510, Mexico; 3 Laboratory A-107, Biomedical Research Institute, National Autonomous University of Mexico , Mexico City, 04510, Mexico; 4 Department of Chemical and Biological Engineering, Chemistry and Molecular Biology, Princeton University, Princeton, NJ, USA; 5 Department of Biological Sciences, Illinois Institute of Technology, Chicago, Illinois 60616; 6 Institute of Chemistry, National Autonomous University of Mexico, Mexico City, 04510, Mexico; 7 Facultad de Química, Universidad Nacional Autónoma de México (UNAM), Ciudad de México, 04510, México

**Keywords:** antimicrobials, genome mining, lantibiotics, lanthipeptides, multi-drug resistant bacteria, natural products

## Abstract

Antimicrobial resistance presents a substantial threat to global public health, demanding urgent attention and action. This study focuses on lanthipeptides, ribosomally encoded peptides that display significant structural diversity and hold promising potential as antibiotics. Genome mining was employed to locate biosynthetic gene clusters (BGCs) containing class II lanthipeptide synthetases encoded by *lanM* genes. A phylogenetic study analyzing homologous sequences of functional LanM sequences revealed a unique evolutionary clade of 17 LanM proteins associated with 12 *Clostridium* bacterial genomes. In silico exploration identified nine complete BGCs, including one super-cluster containing two co-localized operons from *Clostridium cellulovorans* 743B, that encode for two new peptides named clostrisin and cellulosin. Each operon was heterologously expressed in *Escherichia coli*. Molecular weights associated with the expected post-translational modifications of the purified lanthipeptide were confirmed by MS–MS/MS analysis for cellulosin, while clostrisin was not post-translationally modified. Both peptides demonstrated antimicrobial activity against multidrug-resistant bacteria, such as a clinical strain of *Staphylococcus epidermidis* MIQ43 and *Pseudomonas aeruginosa* PA14. This is the first report of lanthipeptides from the *Clostridium* genus produced with its native biosynthetic machinery, as well as chemically and biologically characterized. This study showcases the immense potential of genome mining in identifying new RiPP synthetases and associated bioactive peptides.

## Introduction

Antimicrobial resistance (AMR) is a significant public health challenge. Only in 2019, there were 4.95 million deaths associated with AMR [1], a number expected to increase exponentially. One fundamental objective of the Global Action Plan on Antimicrobial Resistance by the World Health Organization (WHO) is the investment in developing new drugs, diagnostic tools, vaccines, and other interventions [2]. In this context, many antibiotics are derived from bacterial natural products (NPs), which have proven to be a valuable source of antimicrobial agents. During the latter part of the 20th century, the discovery of NPs was hindered by the limitation of traditional methods, which often led to the rediscovery of previously identified NPs. Next-generation whole genome sequencing technologies have a newfound ability to explore and identify biosynthetic gene clusters (BGCs) responsible for NPs production. This renewed focus offers the advantage of preventing redundant discovery and predicting novelty, resistance, and bioactivities. Furthermore, the increasing availability of genomic data has led to the development of bioinformatics tools, such as AntiSMASH [3], Bagel4 [4], and RiPPMiner [5], that have emerged to streamline the process of exploring and discovering BGCs in bacteria, known as genome mining.

Genome mining has emerged as a crucial research area in discovering novel antimicrobial compounds [6–7]. Due to its unique position between basic and applied research, it has become an essential tool for identifying compounds with potential therapeutic applications. Genetic and genomic data mining enables the study of the evolution of genes and genomes across diverse species and populations. This provides insights into the origins and evolution of genes that have evolved as "tools" in the ongoing biological battles in the microbial world. The biodiversity of specialized metabolites is strain-specific, meaning that even closely related organisms can have distinct metabolic capabilities. Consequently, researchers can unveil new clusters and metabolites by tracking the conserved genes participating in biosynthetic processes.

In recent years, notable achievements have been seen in genome mining in discovering antimicrobial compounds. For example, the discovery of teixobactin [8] has paved the way for developing antibiotics with innovative mechanisms of action. Similarly, the discovery of halicin, a molecule identified through a machine learning-based approach, has shown promise as a broad-spectrum antimicrobial agent including some resistant to existing antibiotics [9]. Thus, peptides, including ribosomally synthesized and post-translationally modified peptides (RiPPs), have been regarded as important sources of antibiotics, both historically and through recent discoveries [10].

Lanthipeptides are a class of RiPPs. Lanthipeptide biosynthetic genes have undergone complex evolutionary processes that have produced chemically diverse active peptides [11–12]. These genes are predominantly found in bacteria and have evolved through selective pressures driven by competition for resources and defense against predators [13]. The resulting peptides exhibit unique structural features due to thioether bridges between dehydrated serine or threonine (Dha/Dhb) and cysteine (Cys). These thioether bridges lead to cyclic peptides with the modified amino acid lanthionine (Dha-Cys) or methyllanthionine (Dhb-Cys). The strong antimicrobial activity of nisin, a lanthipeptide produced by certain strains of *Lactococcus lactis,* is dependent on thioether-ring formation [14]. Additional post-translational modifications contributing to their distinct biological activities were identified [15–16]. Intermolecular disulfide bridges were proved to spontaneously form in bovicin HJ50 to exert their natural activity and change spectra of antimicrobial effects when synthetically intramolecularly bound [17–18].

The biosynthesis of mature lanthipeptides involves the translation of *lanA*, which encodes a precursor peptide with two regions: a leader peptide that is recognized by post-translational enzymes and a core peptide where these enzymes produce the formation of Dha-Cys or Dhb-Cys residues. The post-translational modification enzymes involved span different functional domains (a cyclase and a dehydratase), and their activity is tightly controlled at various levels to ensure the production of high-quality peptides [19]. As an example, subtilin production is autoregulated based on the extracellular concentration of the molecule. At the same time, subtilin expression is also controlled by the spaRK operon and the sigmaH (σ^H^) global regulator, which leads to its production during mid-exponential and log-phase [19].

To date, five classes of lanthipeptide synthetases have been identified [20] and are the basis for classifying lanthipeptide gene clusters. For the class II lanthipeptide synthetases discussed in this work, multidomain LanM enzymes catalyze the formation of Dha-Cys or Dhb-Cys [21]. Moreover, *lanPt* encodes a membrane protein with two ABC transporter domains and a C39 peptidase domain. The protein cleaves the leader peptide to generate mature lanthipeptides and exports them to the extracellular environment [22].

The diversity of lanthipeptides produced by these biosynthetic pathways has led to the discovery of many new compounds with potential therapeutic applications during recent years, along with a deeper understanding of their evolution, ecological importance, and gene composition [23]. Lanthipeptides produced by class I and II lanthipeptide synthetases exhibit antimicrobial activity against various pathogens, including drug-resistant strains [23–24]. These molecules are relevant as they have strong antibiotic effects against pathogenic bacteria such as *Staphylococcus aureus*, *Enterococcus sp.*, *Clostridioides difficile*, and *Mycobacterium tuberculosis*. The effects of some of these peptides are attributed to their affinity for the lipid II component of Gram-positive bacterial cell walls [25]. Additionally, there have been reports of lantibiotics such as CMB001 displaying activity against resistant Gram-negative bacteria, including *Acinetobacter baumannii* [26], as supported by the current study. In addition, some lanthipeptides have been shown to have anticancer and immunomodulatory properties [11,27].

A low development of resistance to lanthipeptides has been observed, with nisin being the most well-known case in the food industry. While some strains exhibit resistance due to changes in the cell wall, biofilm formation, or the expression of resistance proteins such as ABC transporters or proteases [28], specific mutations in nisin have rendered previously resistant strains susceptible [29]. The structural diversity of these peptides, coupled with their successful production in *E. coli* [30], has driven research to consider them a source for new antibiotics, as supported by the present study. Identifying the genetic and biochemical mechanisms underlying the production of compounds is of utmost importance in developing effective antibiotics against drug-resistant pathogens. Although certain RiPPs with activity against *Clostridia* have been described [31], the characterization of complete gene clusters encoding for lantibiotics, using their native biosynthetic machinery and not that of nisin or of other non-native peptides, as well as the chemical characterization of the NPs that are produced and the modifications they suffer, from a *Clostridium* genus, have yet to be accomplished. This is due to the difficulty in isolating and maintaining such strains in the laboratory and the limited availability of genome sequences compared to other phylogenetic groups, with published sequences primarily focusing on pathogenic strains [32–34]. Despite these challenges, the study of environmental *Clostridium* has already yielded several interesting NPs, including polythioamides such as closthioamide, a new chemical class from *Clostridium cellulolyticum*, and clostrubin, a polyphenolic polyketide antibiotic and the first reported polyketide from anaerobic bacteria [35–37]. Therefore, the discovery of a lantibiotic with a *Clostridium* origin, reported in this study, has significant implications for developing novel antibiotics. The unique chemical structures of these lantibiotics make them promising candidates for treating drug-resistant pathogens, and the characterization of these compounds from *Clostridium* provides an opportunity to develop new antibiotics.

## Results and Discussion

### Genome mining of LanM enzymes’ sequence diversity to localize novel evolutive clades

**Figure 1:**
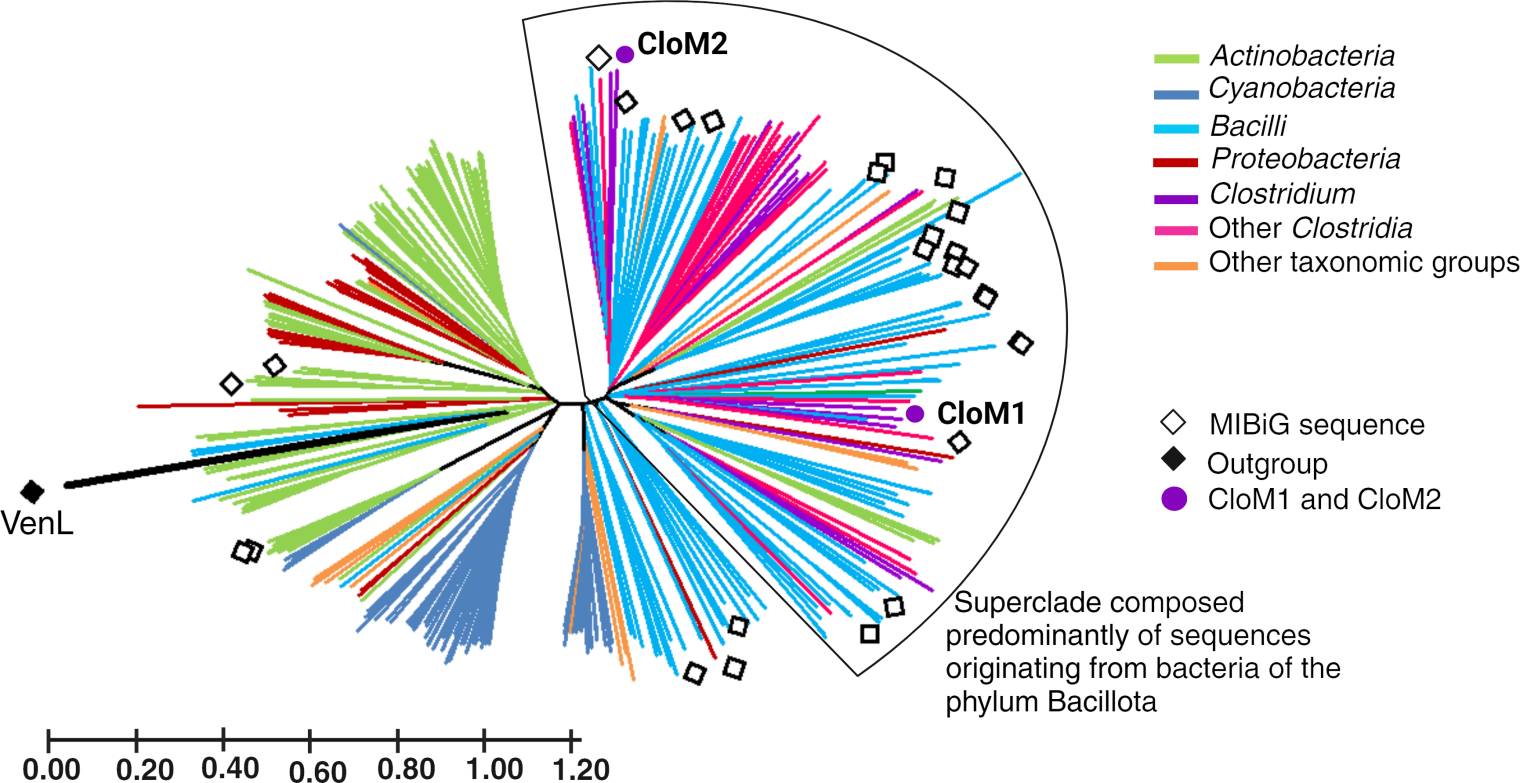
Phylogenetic trees of the LanM synthetase amino acid sequences. Unrooted phylogenetic tree of all the homologous LanM sequences identified by BLAST in our study and those in MIBiG (previously characterized). This analysis involved 343 amino acid sequences; there were a total of 1964 positions in the final dataset. The phylogenetic tree was built from the alignments using the Neighbor-Joining [43], and the evolutionary distances were calculated using the Poisson correction method. A LanL class IV lanthionine synthetase from the venezuelin cluster [44] was used as an outgroup. Square symbols refer to previously characterized LanM sequences from the MIBiG database.

This study used 28 amino acid sequences of LanM enzymes from MIBiG [38] whose activity has been experimentally proven [24] to identify homologous sequences using BLASTP [39] (in BV-BRC [40]). They are considered our reference sequences. Two of these reference sequences are part of the biosynthetic machinery for the production of ruminococcin A [41] and Flv peptides [42] from the clostridial class. As a result, 315 homologous proteins were identified and included in the comparative phylogenetic study. A phylogenetic tree was built (Figure 1) with the reference LanM enzymes amino acid sequences from MIBiG and the homologs identified in our study. The taxonomy of the bacteria to which they belong is indicated in the figure. The selected superclade highlighted in Figure 1, containing sequences that mainly belong to Bacilli and *Clostridia*, was of particular interest because of the novelty that lanthipeptides from *Clostridium* could constitute, since no such BGCs with this taxonomic origin had been described before. The tree also indicates the presence of other yet unexplored LanM clades, such as from Bacilli, Cyanobacteria, and Actinobacteria, which could be interesting to study further due to their sequence uniqueness.

The sequence for venezuelin class IV lanthionine synthetase (VenL – square black symbol) was used as an outgroup, due to the common presence of a LanM domain. The phylogenetic distance confirms the usefulness of the outgroup, confirming a higher similarity within the sequences resulting from the BLAST search.

### Identification and selection of CloA1 and CloA2 BGCs

**Figure 2:**
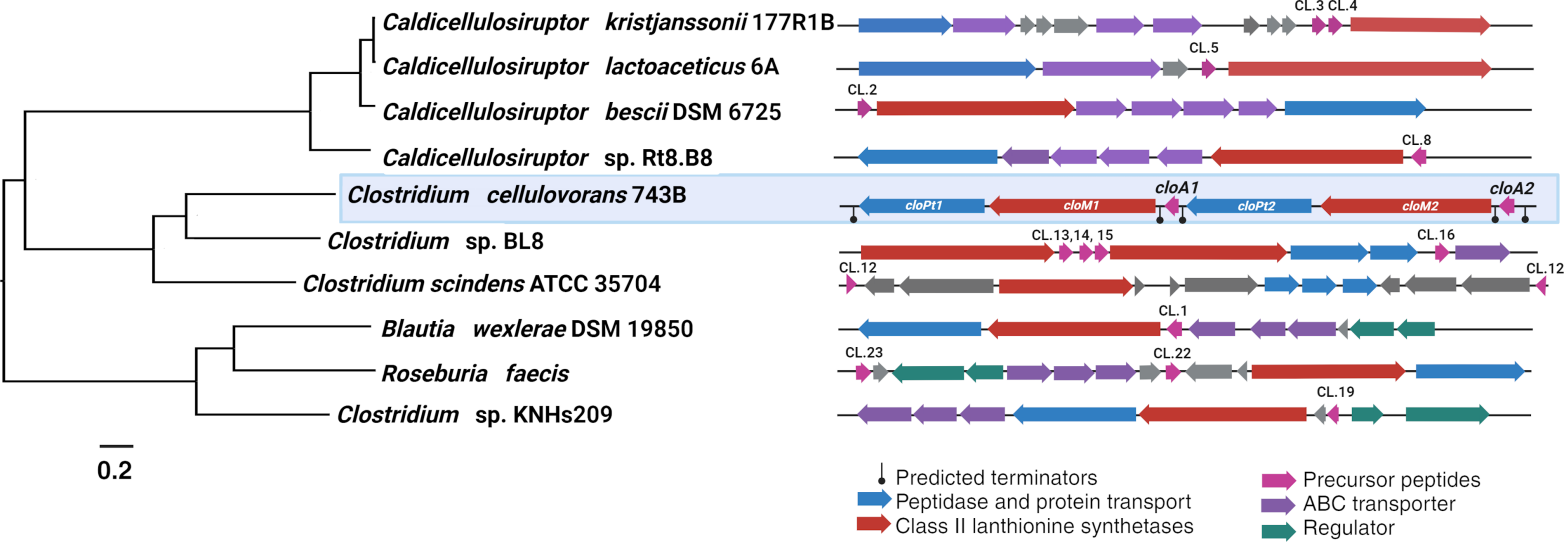
The phylogenetic tree was built by concatenating 1000 shared clostridial genes (left) between the studied genomes, and the lanthipeptide class II synthetase clusters from the selected BGCs of this taxonomic clade are presented (right). The highlighted cluster was chosen for heterologous expression.

For *Clostridia*, there were 37 predicted BGCs (15 of these with at least one precursor and one biosynthetic gene, Table S2 in Supporting Information File 1), with 76 precursor peptides associated. Of these, 2 LanM enzymes and 5 precursor peptides have previously been studied. We decided to focus on the results of the *Clostridium* genus, as there are no described lanthipeptides or associated gene clusters from this taxonomic group. The homology search for novel LanM biosynthetic enzymes from the *Clostridium* genus encountered 17 protein sequences associated with 12 genomes. After analyzing these genomes with AntiSMASH [3], we identified 150 different BGCs of specialized metabolites from different biosynthetic classes. The RiPPs accounted for 36% of these (54 clusters). A total of 14 BGCs containing homologs for class II lanthipeptide synthetases were identified, which in turn helped determine the presence of precursor peptides, gene duplications, paralogues, transporters, and resistance genes in these clusters. Three of these clusters contain more than one biosynthetic gene and one or several precursors, forming superclusters. The RiPPMiner platform [5] was used to predict in silico post-translational modifications of the mature lanthipeptides. Based on these predictions, several clusters did not contain precursor peptides with a predicted lanthipeptide heterocycle formation and were discarded. As compared to reference sequences from MIBiG, no known amino acid precursor sequences similar to previously characterized ones were detected. From these BGCs, 9 precursor peptides (out of a total of 32 precursors) were considered noteworthy of further investigation, based on their physicochemical characteristics, number of predicted cycles (one cycle or more was required), the presence of serine (Ser) and threonine (Thr) residues, and the characteristic cleavage site of the C39 peptidases domain (GG or GA) (Table S2 in Supporting Information File 1) obtained in RiPPMiner [5]. The nine biosynthetic clusters or superclusters these precursor peptides belonged to contained all the genes *lanA, lanM*, and *lanT*. These genes were found to be the minimal machinery required for lanthipeptide biosynthesis (Figure 2). Notably, transporter genes and regulatory elements were not considered in the second filter as their presence is not needed for biosynthesis, albeit it is for resistance. As a result, the *C. cellulovorans* 743 clusters were chosen because they were thought to be complete, novel, and most interesting, primarily due to the presence of two different, apparently independent operons, each with its lanthipeptide precursor (Figure 2). A cluster analysis using BAGEL4 [4] confirmed the presence of terminators. The sequence information for CloA1 (precursor peptide), CloM1 (biosynthetic enzyme), CloPt1 (peptide domain), CloA2 (precursor peptide), CloM2 (biosynthetic enzyme), and CloPt2 (peptide domain) and the associated genes are reported in Table S1 (Supporting Information File 1). The mature peptides formed after the enzymatic removal of the leader peptide by the CloPt domain are called clostrisin (for CloA1) and cellulosin (for CloA2).

**Figure 3:**
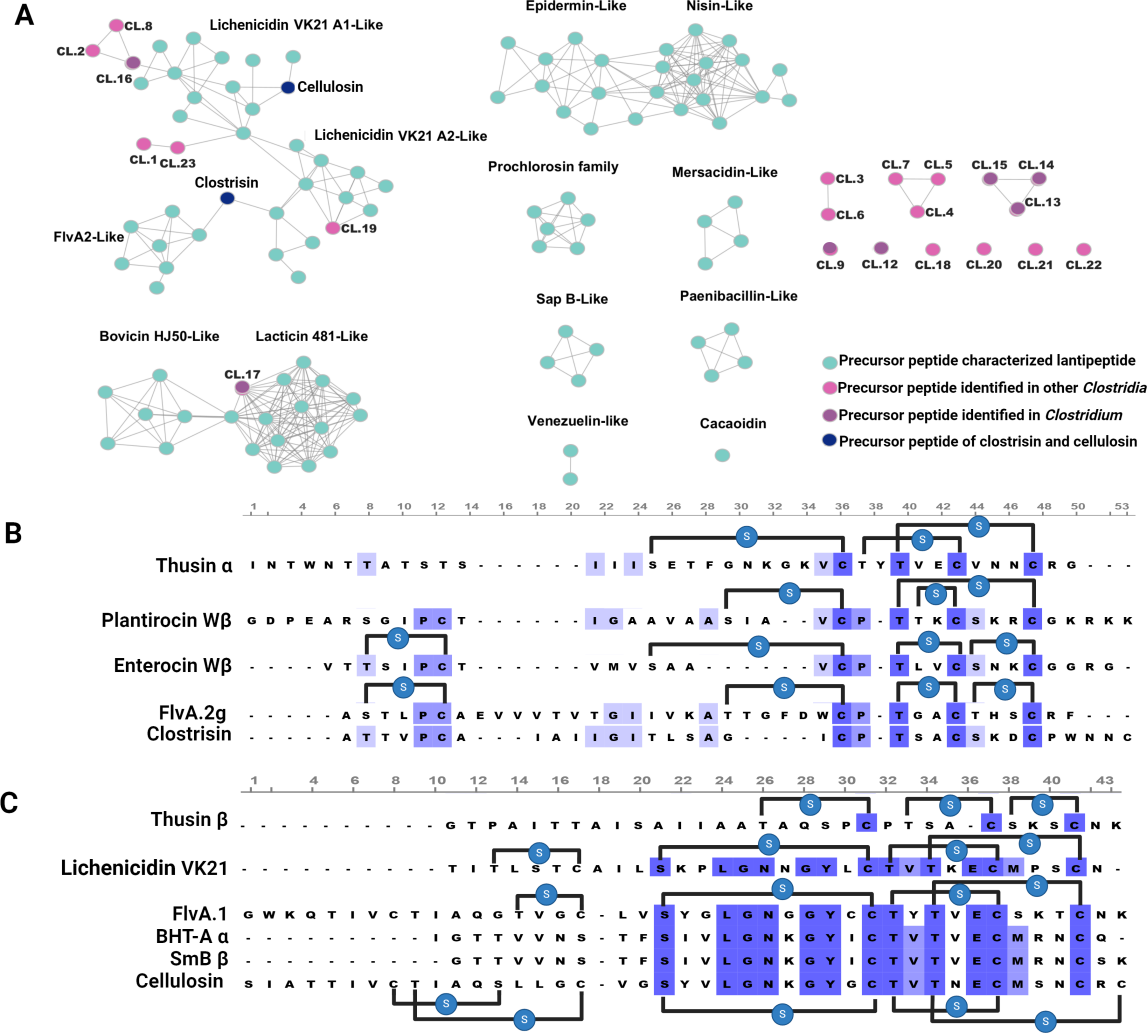
A. Similarity network created with the ESI web tool with the precursor peptide amino acid sequences. In the similarity network of precursor peptides, each blue node represents a characterized lanthipeptide, and each pink node represents the precursor peptides identified in this work. Each line represents a minimum of 27% of sequence identity (EFI – enzyme similarity tool [51]). B. Alignment of clostrisin with FlvA.2g [43], enterocin Wβ [44], plantaricin Wβ [45], and thusin α [46]. C. Alignment of cellulosin with lichenicidin A1 [47], FlvA.1 [43], FlvA.2 [43], BhtA α [48], SmB β [49], Smb α [49], and thusin β [46]. The lanthionine bridges placed on clostrisin and cellulosin are based on the experimental results of the MS^2^ analyses.

To confirm the novelty of the lanthipeptides from the cluster of *C. cellulovorans* 743, clostrisin and cellulosin were compared with known lanthipeptides, generating a similarity network made using the percent identity between the precursor peptides from all experimentally characterized lanthipeptides to date (Figure 3A). We found it noteworthy to examine the two peptides due to their relationship to known lanthipeptides. Despite being part of a diverse cluster of characterized lanthipeptides, these peptides maintain enough sequence divergence, having an identity percentage of no more than 60%. One of these peptides, clostrisin, occupies a hinge position between two divergent lanthipeptide clusters – one represented by lichenicidin and Flv peptides. The other peptide, cellulosin, is located next to belongs to the lichenicidin family. However, it has undergone a significant number of functional mutations. Studying these mutations can provide added data on function–structure relationships and how they impact biological activity. Since we have not found evidence of horizontal transfer of the *cloA1* and *cloA2* gene clusters, it is an interesting speculation that they could constitute the origin of this family and that, by duplicating one of these clusters, *C. cellulovurans* has directed their evolution to serve completely different biological activities.

The amino acid sequence of the precursors shares some similarity with the characterized peptides: for clostrisin with FlvA.2g [42], enterocin Wβ [45], plantaricin Wβ [46], and thusin α [47], and cellulosin with lichenicidin A1 [48], FlvA.1 [42], FlvA.2 [42], BhtA α [49], SmB β [50], Smb α [50], and thusin β [47] (Figure 3B). We find that, especially in the first 20 amino acids of the alignment or the first half of the mature peptide, there are significant sequence differences. Of particular interest, cellulosin forms two larger heterocycles (of seven and nine amino acids) in this sequence region, whilst most lanthipeptides only form small ones (up to five) or none at all. As for the second part of the alignment, where most lanthipeptides form various heterocycles, cellulosin shares a very similar profile to liquenicidin VK21, and as the experimental results confirm, despite the in silico predictions, clostrisin does not form heterocycles. These differences in primary and secondary structure could be responsible for the divergent biological activities we encountered.

### In silico characterization of the post-translational modification enzymes

The selected supercluster is predicted to be formed by two adjacent, biosynthetically complete transcriptional units, each with specific promoter and terminator sites (Figure 2) containing two LanM enzymes (CloM1 and CloM2), the precursor peptides CloA1 and CloA2, as well as two transporter protein peptidases, which we named CloT1 and CloT2 (Figure 2). The amino acid sequences of the C39 peptidase domain of CloT1 and CloT2, further identified as CloPt1 and CloPt2, were subjected to BLAST analysis, revealing a strong primary sequence conservation with all homologous sequences from other clostridial species. A phylogenetic tree was made based on this sequence, showing various organisms within the same class (as detailed in Figure S5A in Supporting Information File 1). Additionally, structure models for CloPt1 and CloPt2 were generated using AlphaFold 2.0 [52] (Figure S5B and S5C in Supporting Information File 1) and compared to the protein PCAT1 [22] (the closest homologous protein with a structure resolved by crystallography, here presented in complex with its peptide ligand (PDB 6V9Z). The amino acid sequence identity between CloPt1 and PCAT1 was 29%, while for CloPt2, it was 24%. Structural alignment revealed RMSD values below 3 Å, meeting the minimum criteria for structural conservation (Table S3, Supporting Information File 1). Furthermore, the catalytic residues within the peptidase domains were confirmed. CloPt1 catalytic residues are Cys35 and His111; for CloPt2, they are Cys18 and His 92. These residues maintained a distance and structural positions like the catalytic residues in PCAT1 (where the catalytic residues are Cys21 and His99).

A similar procedure was carried out for CloM1 and CloM2, leading to the identification of the closest sequences within the clostridial class (Figure S6 in Supporting Information File 1). Structure models for CloM1 and CloM2 generated using AlphaFold 2.0 (Figure S6B and S6C, Supporting Information File 1) were compared with the CylM [53] protein (PDB 5DZT), as it remains the sole LanM enzyme characterized through crystallography to date. The amino acid sequence identity between CloM1 and CylM was 23%, while for CloM2, it was 24%. Structural alignment was performed, and the obtained RMSD values were below 3 Å (see Supporting Information File 1, Table S4). Our findings suggested that these clusters and their associated biosynthetic enzymes hold promise as novel entities with untapped bioactive potential.

### Expression and purification of the CloA1 precursor peptide, CloA2 precursor peptide, and C39 peptidase domain

We could not access the producing microorganisms, so the gene sequences reported in public databases (Supporting Information File 1, Table S1) were synthesized de novo, cloned, and checked by sequencing using a service (GenScript). First, the clostridial nucleotide sequences were codon adjusted for expression in *E. coli* using the GenScript tools, cloned in the pRSF-Duet vector in the MCS1 using the restriction enzymes BamHI and SpeI, and verified by sequencing (GenScript). To express the biosynthetic machinery for the two lanthipeptides, we transformed the *E. coli* strain NiCo21(DE3) (NEB) with the following vectors: pRSF-Duet_CloA1_CloM1, pRSF-Duet_CloA2_CloM2, and pRSF-Duet_CloPt1 (containing the C39 peptidase domain from the clostrisin cluster). The latter contains the peptidase domain of CloPt1, as the complete ABC transporter was deemed too large to synthesize and express in this system. This strategy was functional in previous studies and retained catalytic activity without the rest of the transporter domains [49]. As in the previous literature report, we could confirm that the precursor CloA2 could be cut by the non-native peptidase CloPt1, likely due to this domain's sequence and structure conservation.

SDS-PAGE gel electrophoresis confirmed the expression of all proteins. Each of the LanM enzymes was co-expressed with the corresponding precursor peptide in the same *E. coli* strain (expected size 150 kDa). The precursor peptides and the peptidase domain of CloPt1 were fused with a 6xHis tag at their N-terminal end for purification purposes (Figure S7 in Supporting Information File 1). After purification, we estimated mature peptide and peptidase yields based on SDS-PAGE densitometry between 0.8 and 1.5 g/L of culture for all products. The heat liability of the peptide products challenged the purification, and when trying to perform several purification cycles to increase their purity, we obtained only meager amounts of the peptides. This issue greatly limited the quantity and purity of peptides available for the biological assays.

### The activity of the C39 peptidase domain cleaving the leader peptide of CloA1 and CloA2 precursor peptides

The proteolytic activity was monitored by mixing the purified samples of the C39 peptidase domain with purified CloA1 precursor peptide and CloA2 precursor peptide. The SDS-PAGE electrophoretic pattern confirmed the proteolytic reaction (Figure S7D, Supporting Information File 1). This was further confirmed through HPLC–MS–MS/MS analysis.

#### Mass spectrometric analysis of the CloA1 and CloA2 precursor peptides

**Figure 4:**
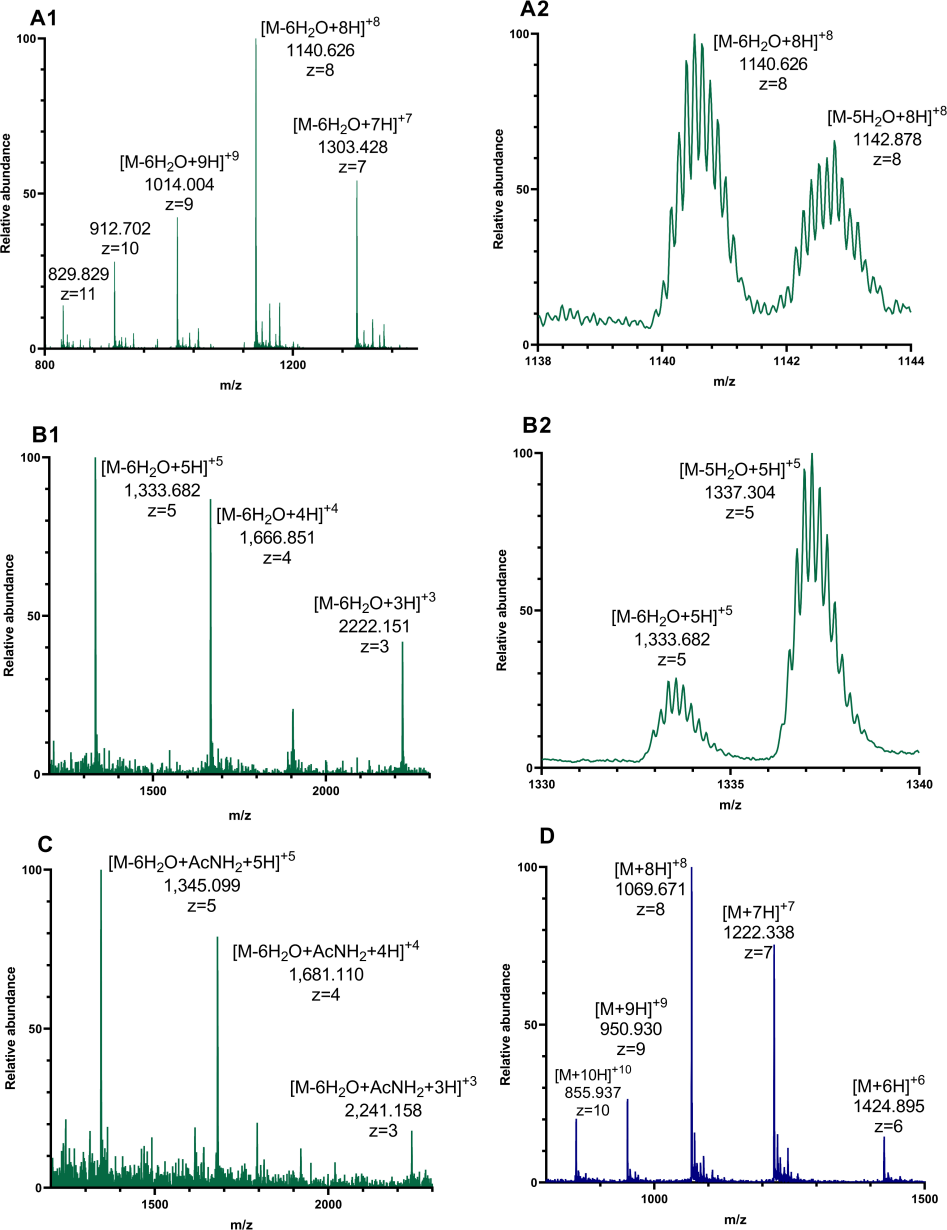
Mass-spectrometric analysis of purified clostrisin and cellulosin (ESIMS spectra). A1 and A2: CloA2, the precursor peptide of cellulosin with a 6xHis tag, was analyzed. A1 shows the ion pattern of CloA2. A2 displays the specific ions with 8 charges of CloA2. GSSHHHHHHSQDPNSSSARLQKNYEELFNEVNENASLQAELNGGSIATTIVCTIAQSLLGCVGSYVLGNKGYGCTVTNECMSNCRC, average mass: 9225.12 Da − 6H_2_O 9147.12 Da. B1 and B2: The tryptic fragment from CloA2 was analyzed. B1 shows the ion pattern of the tryptic fragment. B2 displays the specific ions with 8 charges of the tryptic fragment. C shows the ion pattern of the tryptic fragment after iodoacetamide derivatization NYEELFNEVNENASLQAELNGGSIATTIVCTIAQSLLGCVGSYVLGNKGYGCTVTNECMSNCRC. Average mass: 6771.58 Da − 6H_2_O 663.58 Da + IAA 6720.58 Da. D. CloA1, precursor peptide of clostrisin with 6xHis, was analyzed: GSSHHHHHHSQDPNSSSARLQQNYESKAGFISEMELDELVSNKTVGGATTVPCAIAIIGITLSAGICPTSACSKDCPWNNC. Isotopic mass: 5843.88 Da.

Clostrisin and cellulosin were purified through non-native means using a Ni^2+^ column due to a 6xHis tag at the N-terminal end, followed by elution via imidazole gradient (refer to Figures S7A and S7B in Supporting Information File 1 for SDS-PAGE analysis). The fraction containing the purest products underwent analysis via HPLC–MS–MS/MS experiments. Spectra of the cellulosin sample revealed a pattern of multicharged ions, with the most intense peak at an *m*/*z* of 1,140.64, consistent with the calculated average mass *m*/*z* associated with the CloA2 precursor peptide ion six times dehydrated [M − 6H_2_O + 8H]^8+^ (Figure 4A1 and Table S6 in Supporting Information File 1). Detected MS^2^ ions for the cellulosin experiments are presented in Tables S6–S9 (Supporting Information File 1). Additionally, an ion with an *m*/*z* of 1,142.89 was identified, corresponding to the cellulosin precursor peptide five times dehydrated average mass (Figure 4A2 and Table S6 in Supporting Information File 1). For celullosin, a pattern of multicharged ions was observed, with the most intense peak at an *m*/*z* of 1,068.99, associated with an octuple-charged ion [M + 8H]^8+^ linked to an unmodified precursor peptide average mass (Figure 4D, Figure S4 and Table S9 in Supporting Information File 1). Detected MS^2^ ions for the clostrisin experiments are presented in Tables S10–S13.

Subsequently, a trypsin digestion assay was conducted on the purified cellulosin sample, revealing a pattern of multicharged ions with the most intense peak at *m*/*z* 1,333.71 associated with the fragment N23 to C64 [M − 6H_2_O + 5H]^5+^, in turn, associated with six dehydrations (Figure 4B1 and Table S7 in Supporting Information File 1). Similarly, an ion at *m*/*z* 1,337.31 [M − 5H_2_O + 5H]^5+^ associated with the same fragment five times dehydrated was identified. Cyclization reactions introduced thioether cross-links between dehydrated Thr/Ser residues and specific cysteine thiol groups. However, due to identical masses of dehydrated linear peptides (lacking thioether rings) and cyclic forms (containing Dhb and Dha rings), a derivatization assay with iodoacetamide followed by trypsin digestion was performed for cellulosin, revealing a pattern of multicharged ions with the most intense peak at [M − 6H_2_O^+^AcNH_2_ + 5H]^5+^ with an *m*/*z* of 1,345.16 associated with the fragment from N23 to C64, six times dehydrated, and exhibiting an increase of 57u due to cysteine alkylation (Figure 4C and Table S8 in Supporting Information File 1).

These analyses confirm the activity of the CloM2 enzyme, with the dehydratase domain activity forming a mixture of six and five times dehydrated lantipeptides, and the cyclase domain activity demonstrated by the iodoacetamide derivatization assay, where 5 out of 6 cysteines are involved in the formation of Dha and Dhb along the core peptide, preventing trypsin digestion.

We were unable to detect any modifications of the CloA1 precursor peptide by the CloM1 biosynthetic enzyme, despite previous in silico predictions suggesting that this should be a functional system. This lack of modification could be due to the amino acid sequence of the precursor, which differs from those described in the literature, or to mutations in the CloM1 enzyme. These mutations may require additional co-factors or specific biochemical conditions that we have not yet identified. At this point, based on the information we have gathered, we have been unable to explain the lack of post-translational modification. Although we attempted experiments to mix the CloM2 enzyme with the CloA1 precursor peptide and to mix the CloM1 enzyme with the CloA2 precursor, neither precursor underwent modifications, indicating a certain degree of peptide specificity in the biosynthetic enzymes of this gene super-cluster.

#### HPLC–MS–MS/MS experiment on the CloM2-modified CloA2 tryptic fragment

HPLC–MS–MS/MS experiments were performed on the CloM2-modified cellulosin tryptic fragment, featuring 5 and 6 net dehydrations, followed by the analysis of the sixth dehydrated fragment post-iodoacetamide derivatization to detect possible cross-links and dehydrated residues (Figure 4 and Supporting Information File 1, Figure S1, associated with ion lists in Tables S6–S9). These experiments provided crucial insights into the cellulosin lanthipeptide structure. High-confidence conclusions from the MS^2^ experiment on the fragment with 6 net dehydrations highlighted the linearity of N1–V29. They identified T27 as Dhb, revealing macrocyclization of C30–C39 and suggesting Dhb and Dha residues at T31 and S35, respectively, with potential macrocyclization of S42 forming a lanthionine bridge. Additionally, medium-confidence observations hinted at macrocyclization of C64 (Figure 4 and Supporting Information File 1, Figure S2 associated with ion lists in Tables S6–S9). Furthermore, analysis of the MS^2^ experiment post-iodoacetamide reaction indicated the absence of free cysteines between C30 and C39, supported by alkylated y35 and y24 ions, implying macrocyclization, and proposed the presence of three macrocycles between G41 and the C-terminus (Figure S3 in Supporting Information File 1). Moreover, findings from the MS^2^ experiment on the fragment with 5 net dehydrations suggested one internal tryptic cleavage between G41 and the C-terminus, with K48 as the likely cleavage site if C64 is macrocyclized (Figure S3, Supporting Information File 1).

#### Antimicrobial activity of clostrisin and cellulosin

Antimicrobial activity assays of clostrisin and cellulosin were performed using microplate bioassays with strains from the American type culture collection (ATCC): *S. aureus* 43300 (MRSA), *P. aeruginosa* ATCC 15442 (PA14), *E. coli* IM08B, and *A. baumannii* ATCC BAA 747 as well as clinical strains isolated from patients in Mexico: *S. epidermidis* MIQ43 (multidrug-resistant clinical sample) (internal code from the MicroIQ laboratory library), and *P. aeruginosa* MIQPA25 [54] (multidrug-resistant clinical sample isolated from cystic fibrosis patients), and *C. difficile* R20291. All strains have a multidrug-resistant profile (Table S5, Supporting Information File 1). These bacteria were chosen due to their classification in the ESKAPE group and the species of origin (in the case of clostridioides).

**Figure 5:**
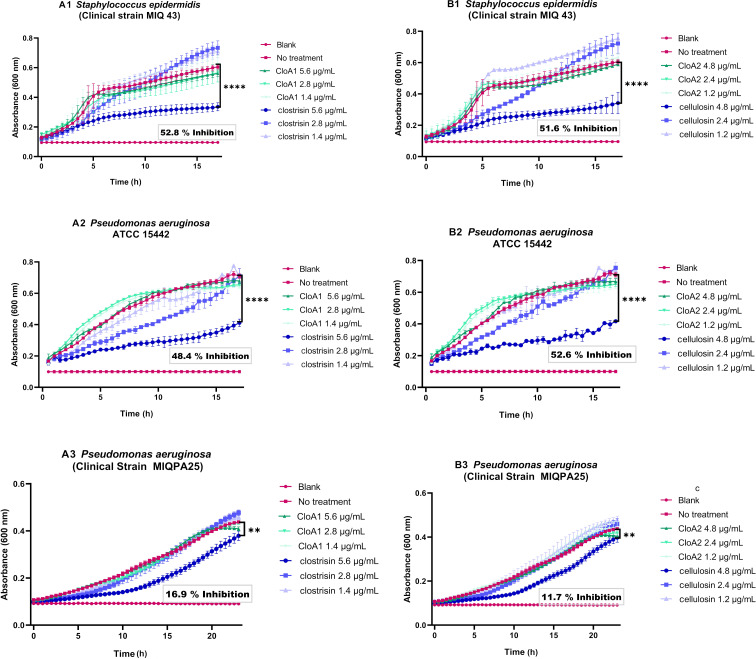
Growth curves of the strains with the bacterial activity of the samples. A. Precursor peptide for CloA1 and clostrisin and B. precursor peptide for CloA2 and cellulosin with the bacteria: 1. *S. epidermidis* MIQ43 and the 2. *P. aeruginosa* ATCC PA14 and 3. *P. aeruginosa* MIQPA25. Tests were performed in sterile polystyrene 96-well flat-bottom-shaped microtiter plates. Inoculum densities were adjusted at an OD_600nm_ of 0.1. The optical density (OD) was measured at 600 nm after incubating the plates at 36 °C for 16 hours at 220 rpm. Three independent experiments were performed, each with three repetitions of every peptide concentration. The statistical significance was calculated with a t-test in GraphPad 8.0.2.

We tested the clostrisin and cellulosin alongside a mixture with the C39 peptidase domain. The approximate concentrations for the mature peptides were roughly estimated based on the SDS-PAGE using densitometry. Each assay was performed at the highest obtainable concentrations: 1.4, 2.8, and 5.6 µg/mL of clostrisin and 1.2, 2.4, and 4.8 µg/mL of cellulosin (Figure 5). We used the same concentrations of precursor peptides in the mixtures of CloA1 and CloA2. In our testing against *C. difficile*, we evaluated clostrisin at 5.6 µg/mL and cellulosin at 4.8 µg/mL. Additionally, to ensure that the observed activity was not due to the C39 peptidase domain protein, we tested these purified proteins on each bacterium at 10.5 µg/mL, where they showed no inhibition.

The peptides exhibited no antibiotic activity against *E. coli* ATCC IM08B, *C. difficile* R20291, *A. baumannii* ATCC BAA 747, and MRSA at the concentrations employed. It was expected that the lack of effect on *E. coli* would occur, as the pre-peptide accumulation did not impact the expression host. This assumption was made due to reports of auto-proteolysis for other lanthipeptides during production in this cell system. This leads us to believe that at least a few mature peptides might have been present. Despite this, the growth curves of the producing strains were not distinct from the control ones, transformed with the empty vector. As a result, it was not surprising that there was no effect on the *E. coli* test strains, which confirmed previous observations.

For *S. epidermidis* MIQ43, *P. aeruginosa* ATCC PA14, and MIQPA25, both clostrisin and cellulosin samples displayed robust bacteriostatic activity at the highest estimated concentration of 5.6 µg/mL and 4.8 µg/mL, respectively, compared to the bacteria grown in LB media (control), and lower but statistically significant effects at lower concentrations (Figure 5). The immature peptides CloA1 precursor peptide and CloA2 precursor peptide showed no discernible effect at the same concentration. These findings highlight a change in the activity after the leader peptide's proteolysis and the presence of mature lanthipeptides (Figure 5). For clostrisin, as it has no post-translational modifications, we expect that the removal of the leader peptide can be the cause for the increase in its biological activity.

Finally, it was extremely interesting to test the lanthipeptides on *C. difficile,* due to the origin of these peptides in the clostridial clade. The strict ecological environments of *Clostridia* are the human and animal microbiota and soil for both anaerobic and aerotolerant organisms. We interpret a lack of effect on *C. difficile,* an intestinal clinical isolate, due to either the presence of resistance mechanisms or the fact that these peptides evolved to be active against soil microbiota members. The habitat of *C. cellulovurans*, the strain of origin of the lanthipeptide clusters, appears to be plant-associated due to its metabolism and isolation environment (wood).

Our results raise numerous questions regarding the ecological functions of the lantibiotics we discovered. Our bioinformatics studies have not provided any clues about the resistance mechanisms of these peptides. Therefore, we plan to conduct further research to gain insights into these functions. This could help us to understand the potential for these peptides to display antimicrobial resistance in the future.

#### Atomic force microscopy

**Figure 6:**
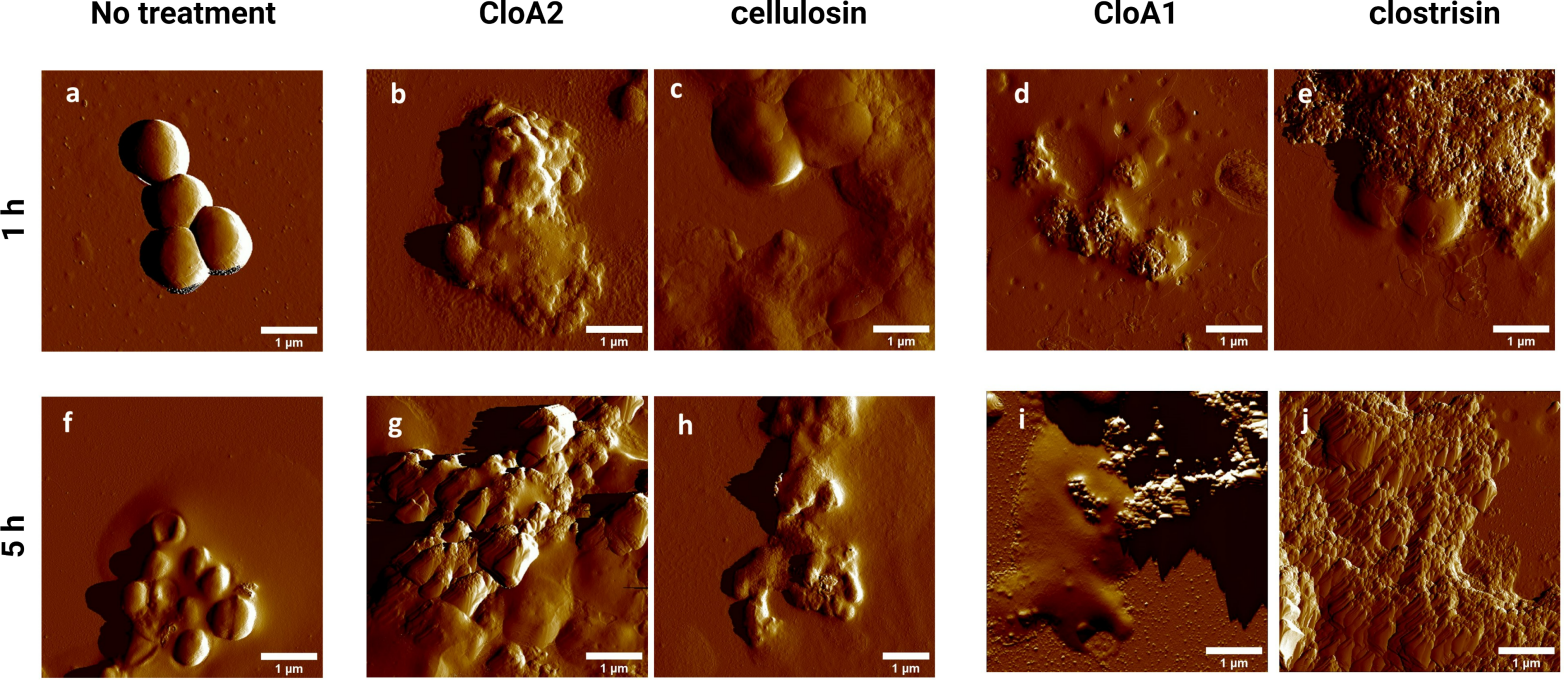
Atomic force microscopy images (peak force mode) of *S. epidermidis* MIQ43 incubated with the different samples for 1 hour and 5 hours. From left to right: no treatment, CloA2 precursor peptide, cellulosin, CloA1 precursor peptide, clostrisin. All samples were evaluated at an estimated concentration of 22.4 ng/µL. Scale bar: 1 μm.

The study employed atomic force microscopy (AFM) in air to observe the bacterial morphological changes on the surface of *S. epidermidis* triggered by the impact of lanthipeptides. The concentrations of peptides were estimated based on the SDS gel band intensity, and we expect there is some uncertainty in these estimations. The samples were treated with an estimated 22.4 µg/mL concentration for 1 and 5 hours. The AFM images of untreated *S. epidermidis* MIQ43 displayed characteristic clustered cocci (see Figure 6A and 6F). The treatment with clostrisin and cellulosin samples resulted in more pronounced morphological changes after 5 hours of treatment. The changes included the loss of the characteristic cocci structure, with the formation of blebs that increased the membrane's rugosity. Besides, cytosolic leakage and remnants of the outer membrane were identified (see Figure 6B, C, G, and H). The treatment with the mature peptides led to numerous blebs visible after 1 hour. Interestingly, no cocci structures were detected after 5 hours of treatment (see Figure 6). The precursor peptides, especially cellulosin, appear to elicit some effects on the membrane of *S. epidermidis.* The increase in peptide concentration can explain the increase in the cellular damage compared with the microplate assay, effects that could not be detected at lower concentrations. Also, the limitation we faced is that, since we were working with only partially purified peptides, our estimates for the concentrations could have a significant error range.

Based on the observations, it can be inferred that both clostrisin and cellulosin directly interact with the membrane of *S. epidermidis*. However, no significant differences were found in the samples regarding using the peptidase C39 peptidase domain. The findings of this study provide valuable insights into the effects of lanthipeptides on *S. epidermidis*, and confirm the previous results acquired during the antimicrobial activity assays. Further studies are needed to confirm the mechanism of action of these lanthipeptides and their molecular targets in *S. epidermidis,* a Gram-positive bacterium.

## Conclusion

The pressing need for new antibiotics has made the search for them a top priority amongst the scientific community. In this study, we utilized genomic mining to forecast the uniqueness of two lanthipeptides derived from the *Clostridium* genus. These peptides were expressed in an *E. coli* host and produced in sufficient concentrations to perform bioactivity assays. The C39-associated domain was purified to facilitate the transformation from pre-peptide to the mature configuration. The mature clostrisin and cellulosin exhibited antimicrobial properties against *S. epidermidis* and *P. aeruginosa* resistant strains and failed to exhibit the same effects against *E. coli* and *C. difficile*. As a result, we present the discovery of two new lantibiotics that have demonstrated efficacy in combating both Gram-positive and Gram-negative pathogens.

## Methodology

### Bioinformatic analyses

#### Lanthipeptides identification

For the search of lanthipeptides, a LanM class II lanthionine synthetase enzyme gene was selected using a MIBiG [38] search of experimentally characterized lanthipeptide BGCs. 28 LanM enzyme amino acid sequences underwent analysis with BLASTP [39] on the BV-BRC platform [40], utilizing the "Reference and representative genomes-Proteins-(faa)" database with a maximum of 100 results and a minimum E-value of 0.0001. All results from each analysis were retained for subsequent studies. The identified homologous LanM amino acid sequences, alongside the 28 experimental MIBiG sequences, were processed in MEGA X [55]. The sequences were aligned using MUSCLE [56] with specific parameters: Open gap: −2.90, Extended gap: 0.00, hydrophobicity multiplier: 1.20, maximum interaction: 16, ensemble method (interactions 1,2): UPGMA, ensemble method (others interactions): UPGMA, and minimum diagnostic length (lambda): 24. Additionally, in MEGA X, phylogenetic trees were made using Neighbor-joining [43]. Evolutionary distances were calculated employing the Poisson correction method, measured in amino acid substitution units per site. Ambiguous positions were removed for each sequence pair using the pairwise deletion option. Bootstrap phylogeny testing was conducted with 1000 replicates, utilizing the Poisson model for amino acid substitution and uniform rates between sites while handling gaps and missing data through pairwise deletion. The genomes associated with LanM enzymes from the clostridial clade were downloaded from the BV-BRCPATRIC platform in FASTA format. Subsequently, the genomes were analyzed using AntiSMASH 6.0 [3] with the following parameters: Detection stringency: relaxed; additional features: all turned on, including BLAST with known sets, BLAST of sets, BLAST of subsets, comparison of MIBiG sets, active site finder, RREF finder, Pfam Cluster analysis, GO term annotation based on Pfam, and analysis with TIGRFam.

#### Precursor peptide analysis

After identifying the lanthipeptide BGCs from the genomes of the *Clostridium* genus, BGCs containing all genes needed for the biosynthesis of a mature peptide were manually selected. The core peptide sequences of the lanthipeptides were then analyzed using the ProtParam and RiPPMiner tools [5] to calculate their physicochemical properties, such as molecular weight, isoelectric point, and GRAVY score (Table S2 in Supporting Information File 1). For the similarity network, a database was created containing 145 precursor peptides of lanthipeptides reported in the MIBiG [38], RiPPMiner [5], and UniProt [57] databases. This input was utilized in the EFI-EST [51] web platform, employing the "FASTA" analysis option with the following parameters: E-value: 0.001; Fragments: disabled. Upon completion of the initial calculation, in the SNN Finishing section, an alignment score threshold of 40% was chosen, with default values for sequence length constraint, and neighborhood connectivity disabled. Network similarity was edited with Cytoscape 3.10.1 [58].

#### AlphaFold models of the biosynthetic enzymes

A phylogenetic analysis was conducted using the closest homologs identified after a BLAST search, employing previously described parameters for CloM1, CloM2, CloPt1, and CloPt2. Additionally, tertiary structure modeling was performed using AlphaFold 2.0 [52] in its CoLab online service [59], with default parameters. Each model underwent energy minimization in Chimera [60]. Subsequently, these models were analyzed in SWISS-MODEL [61] to obtain Ramachandran analysis. Finally, structural alignment was performed using PyMOL for CloM1 and CloM2 with the CylM enzyme (PDB code: 5DZT). For the structural alignment of CloPt1 and CloPt2, the characterized protein PCAT1 (PDB code: 3QF4) was analyzed.

#### Gene expression in *E. coli* and purification of CloA1 and CloA2 precursor peptides and C39 peptidase domain

*E. coli* NiCO21 (DE3) cells were transformed with the following vectors: pRSF-Duet_C39 peptidase domain, pRSF-Duet_CloA1_CloM1, and pRSF-Duet_CloA2_CloM2. The transformed bacteria were cultivated in 1 L LB medium containing 30 mg/L kanamycin at 23 °C. Induction with 0.5 mM IPTG was performed when the OD_600nm_ was between 0.6 and 0.8. Cultures were incubated for 14 h, at 200 rpm, at 18 °C. Subsequently, cells were centrifuged at 10,000 rpm for 30 min at 4 °C. The resulting cell pellets were resuspended in 20 mL of LanA starting buffer (20 mM Tris pH 7.5, 500 mM KCl, 10% glycerol) and lysed by sonication (4.0 seconds on, 10 seconds off, for a total of 10 minutes at 4.0 potency). Finally, the sample was centrifuged for 30 min at 4 °C at 10,000 rpm. Supernatants were stored at −80 °C until purification.

The C39 peptidase domain, and the clostrisin and cellulosin lanthipeptides were linked with a 6xHis N-terminal tag to perform purifications using affinity chromatography with a 1 mL His Trap nickel affinity column. Sonication lysis was performed and centrifuged during 30 min, at 8,000 rpm, 4 °C. The supernatants were loaded into the column. The column was washed with 5 column volumes of LanA wash buffer 1 (20 mM Tris pH 7.5, 500 mM KCl, 10% glycerol, 0.5 mM imidazole), followed by 10 column volumes of LanA wash buffer 2 (20 mM Tris pH 7.5, 500 mM KCl, 10% glycerol, 30 mM imidazole). Finally, the elution was performed with 3 column volumes of LanA elution buffer (20 mM Tris pH 7.5, 500 mM KCl, 10% glycerol, 750 mM imidazole). Purifications were monitored through 20% sodium dodecyl sulfate-polyacrylamide gel electrophoresis (SDS-PAGE) [51]. For HPLC-LC analysis no native purification was performed, for each pellet of 500 mL liquid culture induction of clostrisin and cellulosin lanthipeptides. The cell pellet was resuspended with 10 mL urea buffer (8 M urea, 100 mM NaH_2_PO_4_, 10 mM Tris-base), pH 8.0, frozen at −80 °C, and then thawed at room temperature. The cell lysate was clarified by centrifugation and incubated with 1 mL Ni-NTA resin (Qiagen). The precursor protein was purified using the standard protocol from Qiagen for purifying protein from Ni-NTA resin under denaturing conditions with urea.

Expression was confirmed through Western blot analysis using the 6xHis epitope tag on a lysate sample (Figure S3A, Supporting Information File 1) for the clostrisin and cellulosin lanthipeptides, and the C39 peptidase domain. Affinity chromatography purification (Figure S7B in Supporting Information File 1) was performed for the C39 peptidase domain, resulting in a purity of approximately 96%, based on densitometric analysis of the Coomassie-stained gel. Protein yield was estimated using spectrophotometric quantification. Analysis using the NanoDrop device at 280 nm indicated a 1.25 mg/L yield. For clostrisin, the same analysis revealed a purity of around 41% and a 1 mg/L yield. In the case of cellulosin, the analysis showed a purity of 60% and a 0.8 mg/L yield.

#### Protease activity of the peptidase C39 domain of CloPt1

To optimize the heterologous expression process of protease CloPt1, we refrained from attempting to purify membrane proteins due to technical complexities. Instead, we exclusively focused on cloning the peptidase C39 domain into the pRSF-Duet_C39 peptidase domain vector. The sample containing the C39 peptidase domain, was mixed with samples of CloA1 and CloA2A at a 1:5 molar ratio within a reaction buffer composed of 50 mM HEPES at pH 7.0, 150 mM NaCl, and 5 mM DTT. Cofactors were added as indicated, with the following concentrations: 0.5 mM ATP and 1 mM MgCl2. The reactions were incubated at 4 °C for 36 h. Subsequently, the samples were subjected to analysis using 20% SDS-PAGE.

#### HPLC–MS–MS/MS Experiments

Mass spectra were generated using the Agilent 1260 Infinity II LC coupled with the Agilent 6530 QTOF, with the CID voltage determined by the formula V = 0.036 × (*m*/*z*) – 4.8. Specifically, the MS spectrum of clostrisin and cellulosin was acquired from the non-native affinity chromatography sample. Additionally, the MS^2^ spectra of the N27-C87 fragment with five and six dehydrations were obtained from the trypsin digestion of the cellulosin precursor peptide. Finally, the MS^2^ spectrum of the -K26 to C42 fragment with one alkylation was acquired following an iodoacetamide derivatization reaction and subsequent trypsin digestion. These experimental approaches provide insights into the potential structure of cellulosin.

#### Antimicrobial activity of clostrisin and cellulosin

Antimicrobial activity assays were conducted using the reference strains, *A. baumannii* ATCC BAA 747, *P. aeruginosa* PA14 ATCC 15442 and MIQPA25 (clinical strain), *E. coli* ATCC 10799, *S. aureus* ATCC 43300 (MRSA) and *S. epidermidis* MIQ43. Tests were performed in 96-well polystyrene microtiter plates with bacterial inoculation at an initial OD 600 nm of 0.1. The minimum inhibitory concentration (MIC) for each purified lanthipeptide was determined by testing three different concentrations in three separate wells and repeating the process in three independent experiments. For the highest tested concentration of lanthipeptide (5.6 µg/mL for clostrisin and 4.8 µg/mL for cellulosin) the well contained 178 μL of LB broth, 12 μL of purified lanthipeptide and 10 μL of bacterial culture. The plates were incubated at 37°C for 16 h, measuring the optical density (OD) at 600 nm, under shaking.

The results for MIC and percentage inhibition were averaged. The percentage of inhibition was derived by analyzing the 16 h growth curve data using the standard protocol established in our laboratory, comparing the growth curves obtained across the conducted experiments. All data processing and final figures were generated using Prism GraphPad (version 8.0.2).

#### Atomic force microscopy

AFM characterization was conducted using a MultiMode 8-HR (Bruker). Samples containing *S. epidermis* at an OD_600_ > 1 were incubated with the lanthipeptides at room temperature for 1 and 5 hours. Subsequently, 9 µL of the samples were combined with 1 µL of a 0.1% w/v poly-ʟ-lysin solution in water (Sigma-Aldrich) and immediately deposited onto freshly cleaved mica to be incubated for 10 min at room temperature to allow adsorption. The surface was then rinsed using 600 µL of ultrapure 0.2 µm filtered water and slowly dried using compressed air. Imaging was performed using a Digital Instruments NanoScope V, acquiring 1024 samples per line with silicon nitride cantilevers possessing a nominal spring constant of 0.32 Nm^−1^ and a 0.8–1.0 Hz scan rate. Imaging was conducted at room temperature using the ScanAsyst™ air mode. Images were processed using NanoScope Analysis V.1.80.

## Supporting Information

File 1 Additional Figures and Tables.

## Data Availability

All data that supports the findings of this study is available in the published article and/or the supporting information to this article.
