# Supplementary material for: Discovery of antimicrobial peptides clostrisin and cellulosin from Clostridium: insights into their structures, co-localized biosynthetic gene clusters, and antibiotic activity
Source: Beilstein J Org Chem. 2024 Jul 30;20:1800–16. doi: 10.3762/bjoc.20.159 (PMC11301040; doi:10.3762/bjoc.20.159)
Supplement: File 1 — Additional Figures and Tables. [file Beilstein_J_Org_Chem-20-1800-s001.pdf]

BEILSTEIN JOURNAL OF ORGANIC CHEMISTRY

Supporting Information

for

Discovery of antimicrobial peptides clostrisin and cellulosin from Clostridium: insights into their structures, co-localized biosynthetic gene clusters, and antibiotic activity

Moisés Alejandro Alejo Hernandez, Katia Pamela Villavicencio Sánchez, Rosendo Sánchez Morales, Karla Georgina Hernández-Magro Gil, David Silverio Moreno-Gutiérrez, Eddie Guillermo Sanchez-Rueda, Yanet Teresa-Cruz, Brian Choi, Armando Hernández Garcia, Alba Romero-Rodríguez, Oscar Juárez, Siseth Martínez-Caballero, Mario Figueroa and Corina-Diana Ceapă

Beilstein J. Org. Chem. **2024,** *20,* 1800–1816. doi:10.3762/bjoc.20.159

Additional Figures and Tables

License and Terms: This is a supporting information file under the terms of the Creative Commons Attribution License (https://creativecommons.org/licenses/by/4.0). Please note that the reuse, redistribution and reproduction in particular requires that the author(s) and source are credited and that individual graphics may be subject to special legal provisions.
The license is subject to the *Beilstein Journal of Organic Chemistry* terms and conditions: (https://www.beilstein-journals.org/bjoc/terms)

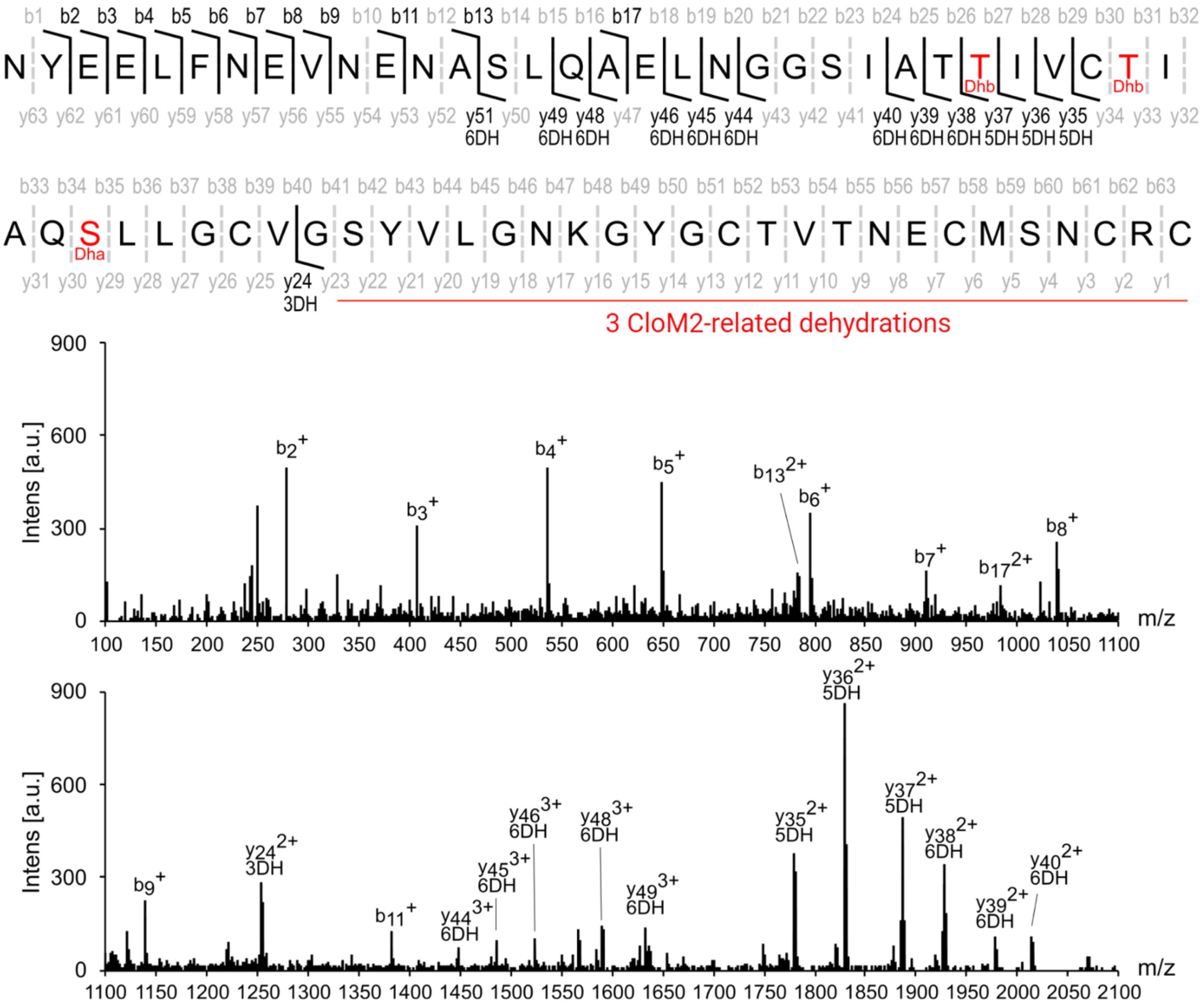

Figure S1. MS^2 experiment on the CloM2-modified tryptic fragment from N24 to C-terminal of the CloA2 precursor peptide harboring 6 net dehydrations.

- Parent ion target *m/z*: 1333.6 ± 1.3 (z=5)
- Collision energy: 43.2 V
- Retention time range: 15.45 – 15.72 min

Supplementary note: High-level confidence conclusions:

1. N1-V29 is linear.
    a. Supporting observation: many *b* and *y* ions observed between N1 and V29
2. T27 is Dhb.
    a. Supporting observation: y_{51}, y_{49}, y_{48}, y_{46}, y_{45}, y_{44}, y_{40}, y_{39}, and y_{38} ions harbor 6 dehydrations, while y_{37}, y_{36}, y_{35} ions harbor 5 dehydrations.
3. C30-C39 is macrocyclic.
    a. Supporting observation: No fragmentation observed between C30 and C39
4. T31 is Dhb, and S35 is Dha.
    a. Supporting observation: y_{37}, y_{36}, and y_{35} ions harbor 5 dehydrations, while y_{24} ion harbor 3 dehydrations. This means there are two CloM2-related modifications

between I28 and G41. T31 and S35 are the only possible residues that can be dehydrated by CloM2.

5. S42 is macrocyclized (i.e. forms a lanthionine bridge).
    a. Supporting observation: No fragmentation was observed past S42. If S42 were not macrocyclized, the most N-terminal residue able to form a macrocycle is C52. Then, S42-G51 would be linear, in which extensive MS2 fragmentation would be expected.
6. (medium-level confidence) C64 is macrocyclized.

Supporting observation: y_1 not observed. If C64 were not macrocyclized, y_1 and y_2 could be observed. C62 might be macrocyclized, likely suppressing y_2 fragmentation. However, a high level of y_1 fragmentation would be expected. Normally, with lanthipeptides, if the last residue is linear, there is a tendency for a high intensity of y_1 ion.

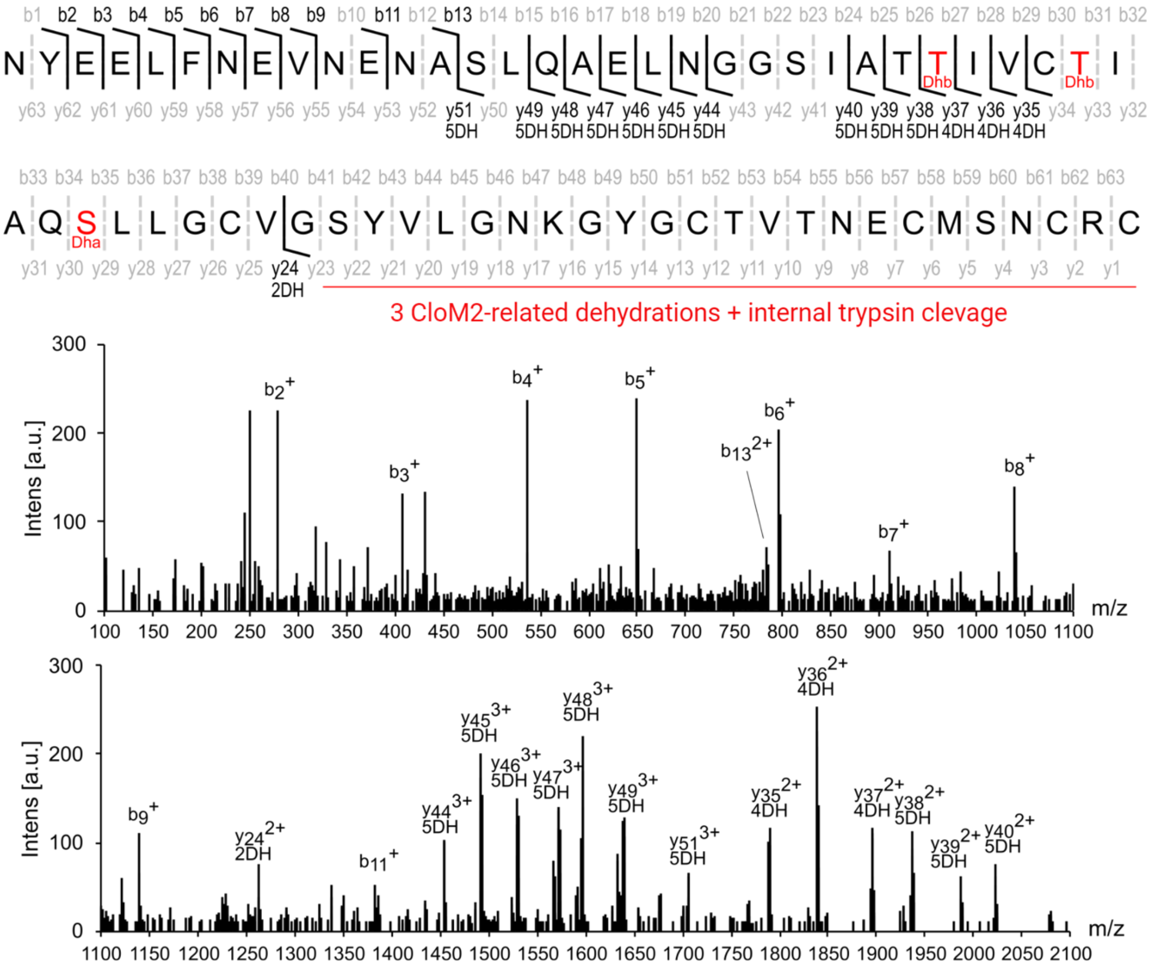

Figure S2. MS[2] experiment on the CloM2-modified the tryptic fragment from N24 to C-terminal of the CloA2 precursor peptide after iodoacetamide reaction, harboring 6 net dehydrations and 1 alkylation.

- Parent ion target *m/z*: 1345.0 ± 1.3 (z=5)

- Collision energy: 43.6 V
- Retention time range: 17.11 – 17.25 min

High-level confidence conclusions:

1. C30 and C39 are macrocyclized.
    a. Supporting observation: y_{35} and y_{24} are alkylated (+57 Da), meaning there is no free cysteine in between.
2. There are three macrocycles between G41 and the C-terminus.
    a. Supporting observation: y_{24} is alkylated. There are four cysteines and 1 alkylation, so one of the cysteines must be free. Three cysteines form (methyl)lanthionine bridges.

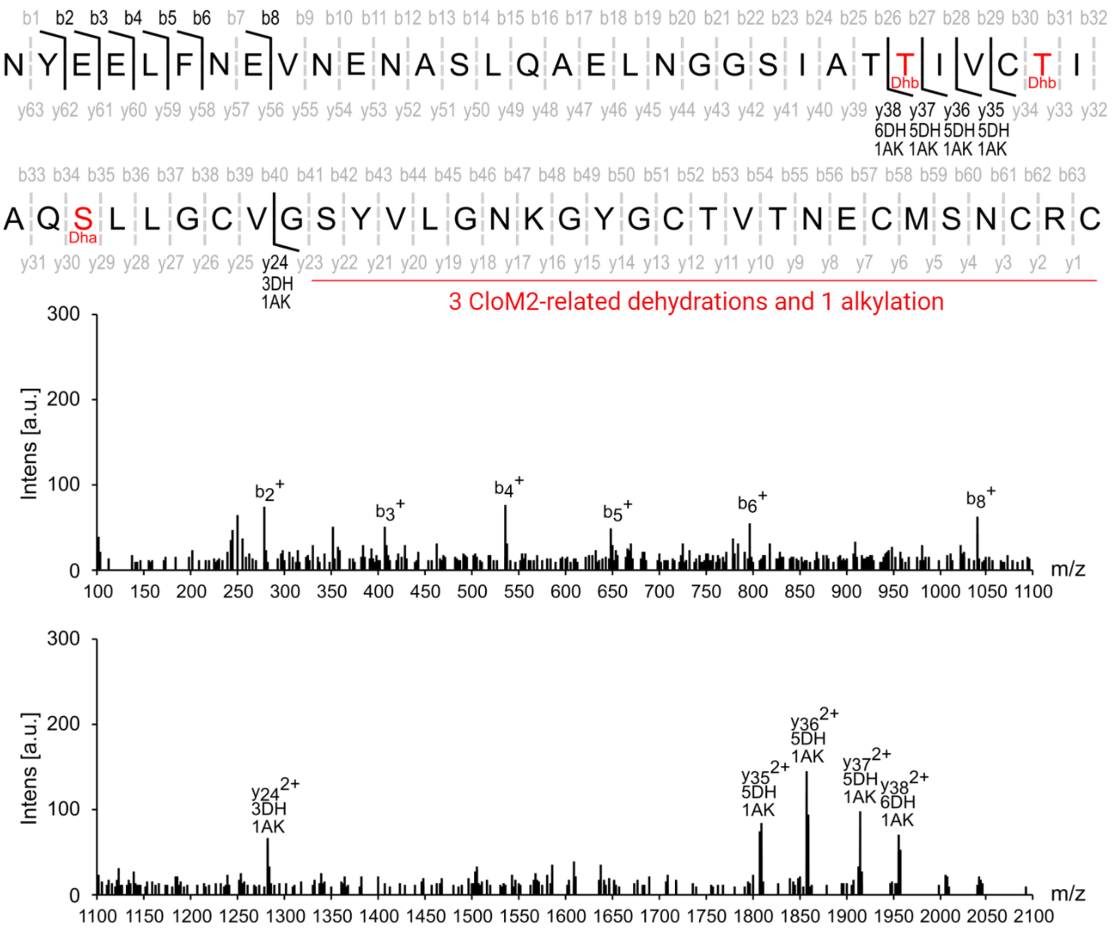

Figure S3. MS[2] experiment on the CloM2-modified the tryptic fragment from N24 to C-terminal of the CloA2 precursor peptide harboring 5 net dehydrations.

- Parent ion target *m/z*: 1337.2 ± 1.3 (z=5)
- Collision energy: 43.3 V
- Retention time range: 14.67 – 15.11 min

Supplementary note 2: Results explanations:

1. The high-level confidence conclusions are the same as from the MS^2 experiment on the 6-fold dehydrated **CloA2 precursor peptide** tryptic fragment.
2. One internal tryptic cleavage between G41 and the C-terminus is suggested. K48 and R63 are possible trypsin cleavage sites. If C64 is macrocyclized, then K48 is the most likely cleaved by trypsin.

Detected MS^2 ions are presented in Tables S6 to S9.

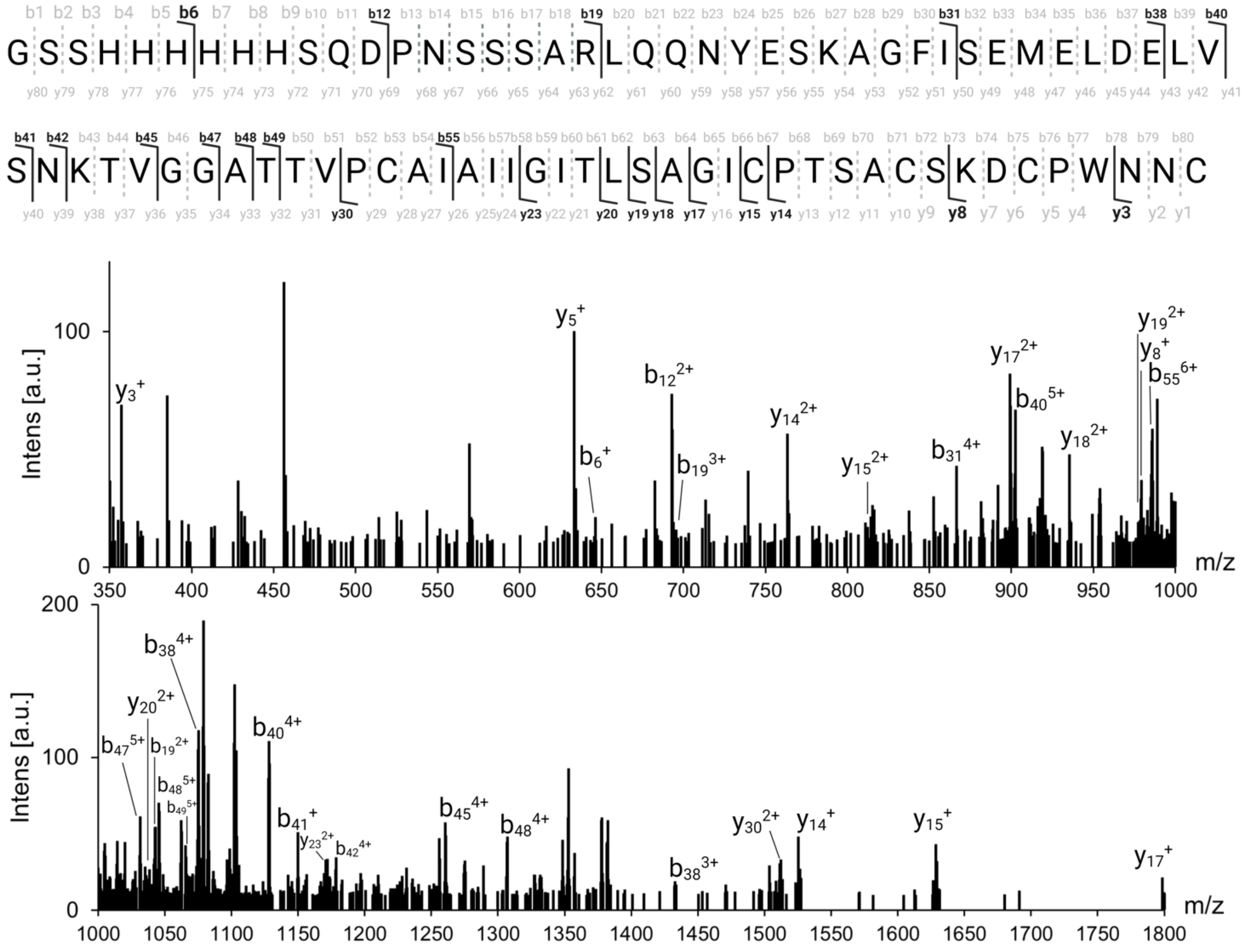

Figure S4. MS2 experiment of CloA1 precursor peptide, linear peptide, with no post translational modifications.

- Parent ion target *m/z*: 1069.0 ± 1.3 (z=8)
- Collision energy: 33.68 V

Retention time range: 14.67 – 15.11 min

Supplementary note 3: Results explanation:

The high-level confidence conclusions are that CloA1 precursor peptide is a linear peptide without posttranslational modifications.

Detected MS^2 ions are presented in Tables S10 to S13.

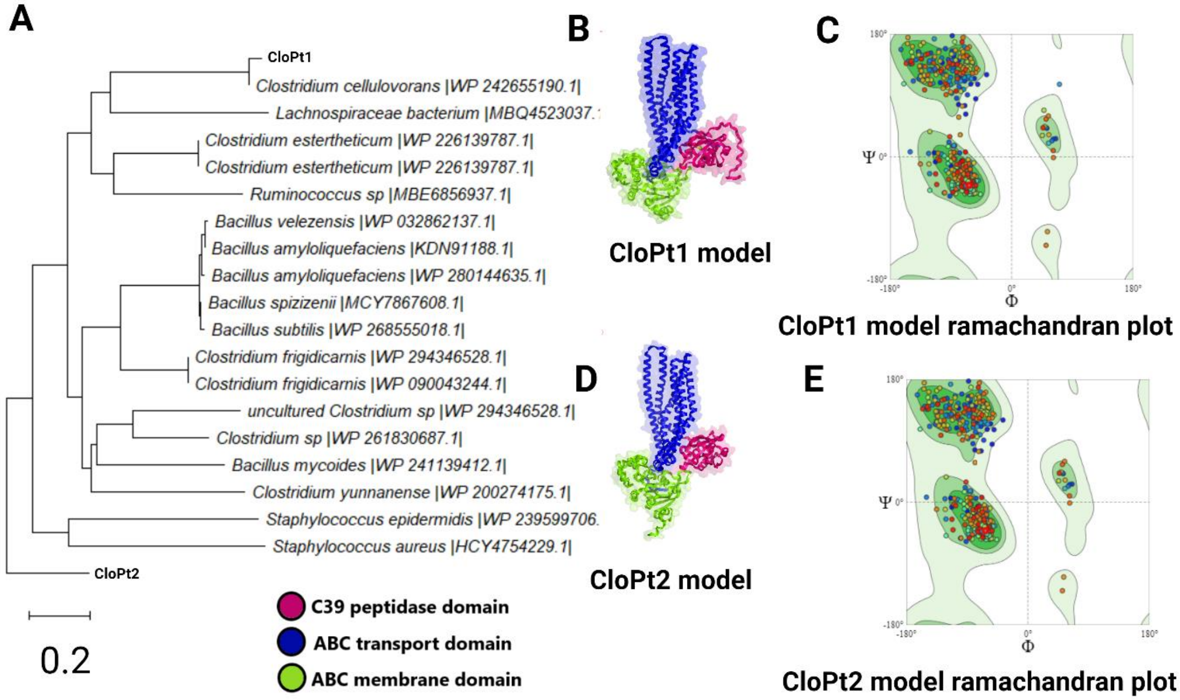

Figure S5. A. Phylogenetic tree with the amino acid sequences closest to the C39 peptidase domains of CloPt1 and CloPt2, models **B** and **D** generated with AlphaFold 2.0, minimized with Chimera, figures **C** and **E**, Ramachandran plots of the models for CloPt1 with favored Ramachandran of 96.85% and for CloPt2 with favored Ramachandran of 96.66%.

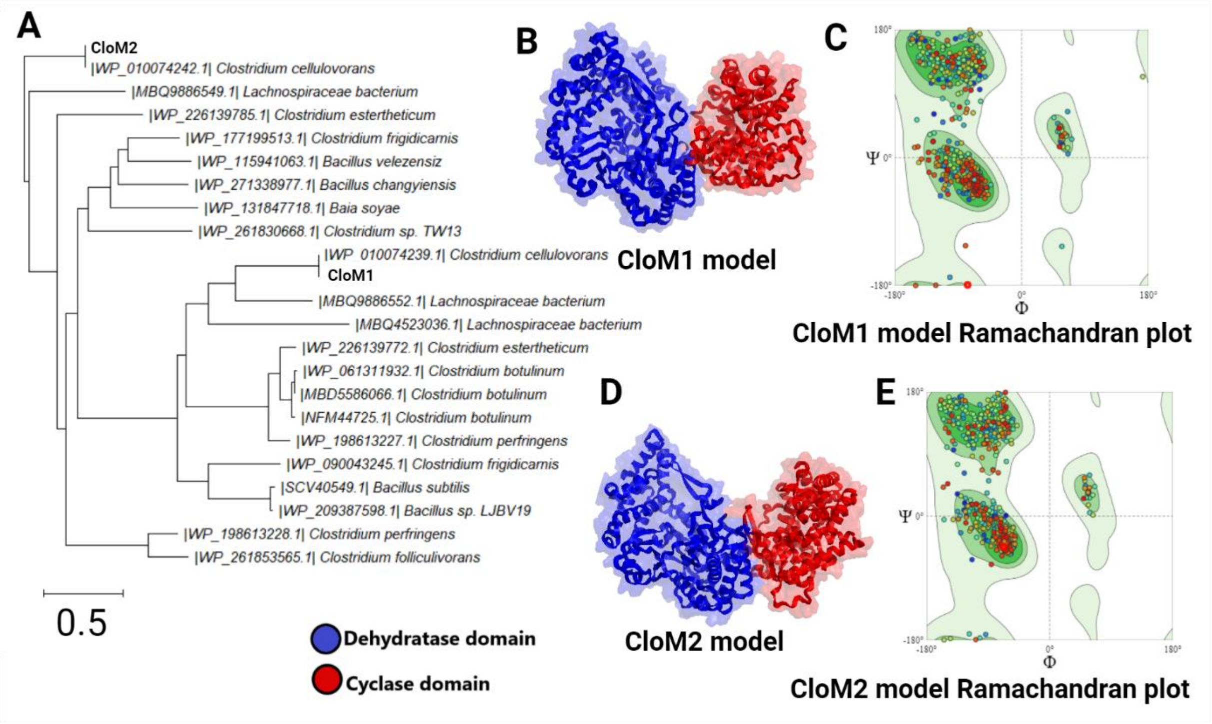

Figure S6. A. Phylogenetic tree with the amino acid sequences closest to the total proteins CloM1 and CloM2, models **B** and **D** generated with AlphaFold 2.0, minimized with Chimera, figures **C** and **E**, Ramachandran plots of the models for CloM1 with favored Ramachandran of 96.93% and for CloM2 with favored Ramachandran of 97.21%.

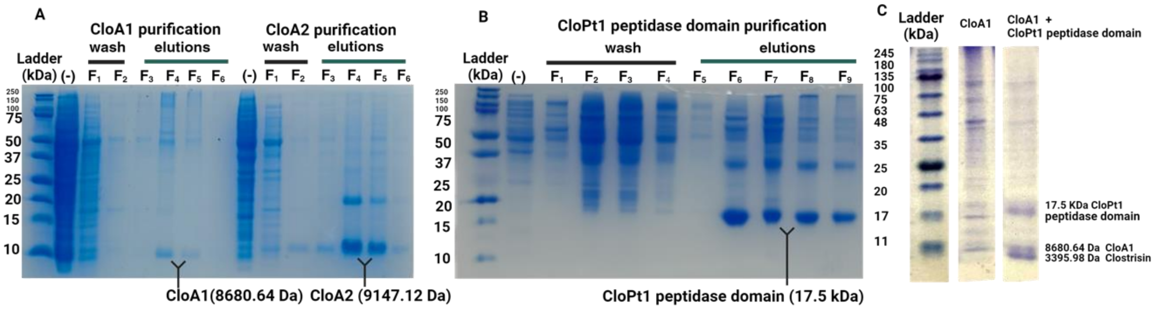

Figure S7. Main product purification SDS-PAGE electrophoresis gels. "F" denotes fractions, and (-) denotes the lysis sample. The non-native and native purification process is described in the methodology section. **A.** 12% SDS-PAGE gel of the nickel affinity non-native purification for CloA1 precursor peptide (8.3 kDa) and CloA2 precursor peptide (9.2 kDa). **B.** 12% SDS-PAGE gel of the nickel affinity native purification for CloPt1 peptidase domain. Fractions from 6 to 9 display lanes demonstrating the presence of the C39 peptidase domain (17.5 kDa). **C.** Proteolytic activity of CloA1 was monitored after 48 hours at 4 °C in the reaction mixtures, as described in the methodology section.

Table S1: Nucleotide sequences for the genes *cloA1*, *cloM1*, *cloPt1*, *cloA2*, *cloM2*, and *cloPt2* and amino acid sequences for the corresponding proteins.

Sequence information in fasta format

> *cloPt1*
ATGAAGTTGTTTGGAATAGATATAACTGAAATATTTTCGCTATCTTTTAGGAAGGAAAAGTATGTTATGAA
AAAGGTCGATATAGTAAAGCAAATATCTCAAACAGAATGTGGGGTATGTTGTTGTGTAATGATTCAAAATT
ATTATAATGTTAATACTTCATTAGGGCAGGTTAGAAAAGAATTAGATGTTGGCCGAGATGGAATGTCAATG
ATTCAGATTTATGAGTACTTAACAAGTAAAGGCTTTTCATGCAAACCCTTCAAGACAAATAATGTTCTTAAG
CTATCTGAGATTACGTTGCCATGCATTGCTTTTTTTGGTGAAAATCATTTTGTTGTTGTTGAATCAATAAAA
AATCAAAAGGTTATTGTCTGCAATCCAAGTATTGGAAGAAAAGTTATGTCAATTCAGGAGTTTGATAAAGA
TTTTAGTGGAATAATTCTACAAGTGTTACCTACAGATAAAGTTGAAAAAGTTTCAAAGAAAGAAAACCCAT
GGCATGTAGTTATTAAGTTGTTAAAAGAAAACAAGCGTTTACTCCTTGTAGCAGTATTATATATGATCCTAG
CATATGCTATATACTTGGGTGTACCCATGTTTGAACAAAATTTGATAGATAAAGTAATAGTTTTATCTAACC
TTGATACAGTCTATATTTTCTCATTAATTTTAGGTTGTTTACTTATTGGCTATGCAATTATTAGCCTTTTGA
GAGGATTTAAGTTATTAACTTTAAATATTAATATAGCTAATAAGATGGAGATTGGAACGTATAATAAATTA
TTACGACTTCCATATAAGTATTTTGAAGTTAGAAATACTGGGGAATTATTGTATAGTTTAATCTGTGTTTCT
TCTGTAAGAGAACTATTAGCTACATATATTGTTAATGGAGTAATAGATGTTGGTGCAGTGATAATAGTAAG
TATATATATGTTTCAGAAGTCATGGTTATTAGGAATTTTTGCTTTAGTTTTATGGATACTAAACGTAGTTTT
TTTATTTTTAATGCAGCCTAAGTTAAGTGGAGTTGTTGATGAAGAATTAGTGCAAAGAGCTACTGCACAATC
GTTACAAACAGAAGCTTTAGGATCAATAATGTCTATAAAAAATGATGGGGTTAGAAAAACAGGTATTAAATG
ATTGGAAAATAGTATATGAAAAAGTAATAAGAAAATTTTCAAGGAGAATTAATATTCAAAATATAATTGGT
GCATTCAATAGTAGTATTAATTTGTTTGGACCAGCATTCATAGTATGCAGTAGCATGGTTTTATATTTTTAT
GGATTACTGTCTATTGGTGAAATAGTTGCTTTTCAAACAATATCATCAATTTTTTTTGGAATTGCAAATAAT
ATATGTACTGCATATTCTCAGTTTATTCTTGCTTCTTCATATTTAGAAAGAGTAGATGAAATTTGGTCTACT
GAAGAAGAAAATTATAATGAAAATGGTATAGCAAAAGATATTAAAGGAGATATTGAAGTCAATGATATAAC
TTTCAGATATTCTAAAACTTCTCCATATGTAATTGAGAAGATTAACTTAAAAATTAAAGCTGGTTCAAATG
TAGCAGTTGTGGGACCTTCTGGTTCAGGAAAAAGCACACTAGGAAAAATTTTAAGTGGTCTATATGATATT
GAAAGTGGAGATATTAATTATGATGGTATCAGCATCAGAGAATATAATAAAAATGAATTGTGTAAAATGAT
AGGCATTGTACCTCAGGAAGTAATGCTTTTTAATAAATCAATTTATCACAATATTGTAATGAATAATGGAA
GTATACCGCTTAATGAAGTAAGAGAAATATGTAAATTGGTATGTATTGATGAAGAAATAATGAGTATGCCA
ATGCAATATAATACTATAATATCAGAAATGGGATTGAATTTATCTGGTGGACAAAGGCAAAGGATACTGTT
GGCACGAATCCTCATCAGTAAACCTAAAATTCTAATTTTAGATGAAGCTACAAGCTCGATAGATACTATAAG
TGAAGAAAAAATATCAAGGTATCTAGCTGATTTAGGATGCACTAGAATTACAATTGCACACCGCTTATCAAC
AATTATAAATGCAGATTGCATCTATGTAATGAATAAGGGAAGAATTATCGAGTCGGGAACGCATAATGAAT
TAATAGAGAATGGTAAGGTATATAATGAGTTGTACTATTCTGGAAACACAGATTAA

> CloPt1
MKLFGIDITEIFSLSFRKEKYVMKKVDIVKQISQTECGVCCCVMIQNYYNVNTSLGQVRKELDVGRDGMSMIQI
YEYLTSKGFSCKPFKTNNVLKLSEITLPCIAFFGENHFVVVESIKNQKVIVCNPSIGRKVMSIQEFDKDFSGIILQ
VLPTDKVEKVSKKENPWHVVIKLLKENKRLLLVAVLYMILAYAIYLGVPMFEQNLIDKVIVLSNLDTVYIFSLIL
GCLLIGYAIISLLRGFKLLTLNINIANKMEIGTYNKLLRLPYKYFEVRNTGELLYSLICVSSVRELLATYIVNGVID
VGAVIIVSIYMFQKSWLLGIFALVLWILNVVFLFLMQPKLSGVVDEELVQRATAQSLQTEALGSIMSIKMMGLE
KQVLNDWKIVYEKVIRKFSRRINIQNIIGAFNSSINLFGPAFIVCSSMVLYFYGLLSIGEIVAFQTISSIFFGIANNIC
TAYSQFILASSYLERVDEIWSTEEENYNENGIAKDIKGDIEVNDITFRYSKTSPYVIEKINLKIKAGSNVAVVGPS
GSGKSTLGKILSGLYDIESGDINYDGISIREYNKNELCKMIGIVPQEVMLFNKSIYHNIVMNNGSIPLNEVREICK
LVCIDEEIMSMPMQYNTIISEMGLNLSGGQRQRILLARILISKPKILILDEATSSIDTISEEKISRYLADLGCTRITI
AHRLSTIINADCIYVMNKGRIIESGTHNELIENGKVYNELYYSGNTD

> *cloM1*
ATGGAAAAGTTGTTTGGAAACTACATTAAAAAATCTTTTGAAATTAAGGAAATAAATAAAATTGTTCATAG
GACTAAAGAATATTTTGGAGAGTTTTCTAGATATTATTTATATATTTTTTATTACGAATTTGACAATGCAA
TAATCAGAAAATATGGTGATTTATTTGATCAATTTAATAGCATAATTCAACAACAATTAGATATATTTTTC

AAGGATATATTTTATATAAGCATAAGAGTGTTAATTGTTGAAATGAATATTATGAAAGATGAAAATAAGCT<br>AGTTGGTGCAAATAGCCAAGAAAGATACTTGTATTATTCGGAACTTTTAACAAAAGAAGAATACATTGATT<br>ACCTAATAAGCAAGTATCCGGTTTTAAATAGAATTTTATTGGAAAAGTGTAAAAATCAAATTAGACTAATT<br>AATGAATGTTTAAGTAATTATATACAAGATTTTGAACCGATGTGTGAAACTTTTGAAATTTCAGCTCAAAG<br>TAAAATTAAGCAAATAATTGTTACATCGGGGGACTCACATAATGGAGGTAAGAAGGTTATCTTATTAGAAC<br>TTTCAGAAAACAAAATATTATATAAGCCTCATGATTTTTCATCAGAAAAAATATTTAATGAAATACTAGAA<br>TCCATAAATAAAGAACAGTGCATAAAGTATAAGTTAAAGACTATTAAGAATATTACTAGGGATAATTATGC<br>ATGGCAGGATTATATCAAAGCAATTGGATGTACGAAGATTCAAGAGGTGGAGGAATATTACTACAAGATTG<br>GAGCATATTTGGCGGTATTGTATTCTTTGGGATGCGAGGATATTCACAAGGAAAATATTATAGCTTCTGGTA<br>ACAATCCATATCTAATTGATATGGAGACACTATCAAATTGTCAGGCACCTTTAATAAATGATAAAGCTACAA<br>TGCTAGAGCACTTTTTTTATGAGAATAGCCAATCAGTCTTCGGAACAATGCTTTTACCTACTAATTCAGCAG<br>TGTCTATATTTGATTATGACATTGGAGGAATCTCTGGAGATGATAACATTGAAACATCAAAATGGGAAGCT<br>TTTGATATAAAAAATCAAGGAACTGATAATTTACAATTTGTTAAGGAATCTAAGTTTATAACTGGTGGATG<br>TGATAACATAGTAAAACTTAATGGTGAGGCTACTCGTGCTAGAGATTATTACAAGAATATTATAGAAGGAT<br>TTTCTGATTGTTATAAAATATTTATAAAAACTCCAAATAAAGTTGTAGATATATTAAAAGAGAGTGAAGTT<br>ATAATACGACAGGTTTTAAGACCAACAGCAGTTTATAGTAAATTTTTAGAAGCTTCTACTTATCCAACTTAT<br>CTAACAAATGAAGAATCTTTTAGAGGTCTCTTTGCAAAGTTAGATAATTTGGAAGAGGTAAAAGAAAAGAA<br>AAAAGCACAAATTGAAATAGATAGTTTATATGAGTTTGATATACCATATTTTTATTCCGATTTAAATACAA<br>CTAACATTTATTCAGTCAAAGGAAAAGTTGATAATTACATAAATTATTCTGTTATGGATGCTATAAGCGGA<br>AAGGCTAGGAAGTTTTCGGAAAAGGATTTAAAGGTGCAAATATATTATATTAACCTTGCACTATCAACCCA<br>ACCTAACACATCTGAGATGATTTATAAATATAATTTATTCAGTAAATCACAAAATTTAAAGAATATTACAA<br>TTAATCATATTGCTAAACAGATAGGAGATGTAATTGAAGAAAAACTAATTTGGGATTATAGAAAACAAAGT<br>TGCTTTTTTATGATTGATACGGTAGTAAAAGAAAAGAGAAAATACTGTAGCATGGATTCTAGTTTGTATGG<br>TTCGGGTGGAGTAATTCTGTACTATCTATCGCTATACAAAGCTACTTGTGATTTGAAGTATAAACATATAGT<br>CGAAGGATTAATTAATGGACATATAGGGTTACATTCAAAAAAATCCTTAGAGGAGGGGATAGGATTATTTT<br>CAGGGTTAACTTCATTAGCATATATATACTATCAATGTTATAGGGTTTTAGGAAAGAAACAGTATTTAGAA<br>GATGTCTTAGAAGTTCTAAAGGAAATAGAAGAAAATTTACATATGTGGGATGAGCTAGATTTATCAACAGG<br>GTTATCAGGTGTTATCATATTTCTATCAGAACTTTATAAAACAGAAAATAATGAACAGTATCTATTAATTG<br>CAAATGGCTTAGCTGATAAATTATATAAATTAGTGTTGAAAGCTAATTTTAATATGTTAACAGGATTAGCA<br>CATGGATATGCAGGAGTTGCTTGGGCTTTATATAGTATAGGGCATATAGTTAATAATGAGGATTATATTAA<br>TTGCGCTATGAAATGCATTGAAGAGGAAAATATGTATTATGATTCAGAAAAAAACAACTGGAAAGATAAAA<br>GAAATGGTAGTTCAGAAAGCTTCTATTGGTGTTATGGTGCTTTTGGGATAGGTATCGCAAGATTAAAGATG<br>TATGAAATAGCTCATAATGATATTTTATTAAAGGATATTGAAAAATGCAGATATATATATATTAATTATAA<br>TTTAATTAATAGTAAGTATAATCATTCTTTATGCCATGGGCTTACAGGAAACTTATCAGCAATAAAGATGTT<br>TAGTGATTTTTATAAGAATGACAATAAGTTAAAAAAGAAATATAAGGAAATGTTGGATATATTATTAAATG<br>ATGTTGTAAATGGCAAAGTTATATGGGGTGATAAGGAGCTCATAGAAGATTATTCATTTATGATTGGTTTA<br>TCGGGTATTGGGTATGAGTTATTAAGGCACGAAGCTACAAACTCTGTAAATATTTTAGCATTAGAAGTGTA<br>A
**> CloM1**<br>MEKLFGNYIKKSFEIKEINKIVHRTKEYFGEFSRYYLYIFYYEFDNAIIRKYGDLFDQFNSIIQQQLDIFFKDIFYIS<br>IRVLIVEMNIMKDENKLVGANSQERYLYYSELLTKEEYIDYLISKYPVLNRILLEKCKNQIRLINECLSNYIQDFE<br>PMCETFEISAQSKIKQIIVTSGDSHNGGKKVILLELSENKILYKPHDFSSEKIFNEILESINKEQCIKYKLKTIKNIT<br>RDNYAWQDYIKAIGCTKIQEVEEYYYKIGAYLAVLYSLGCEDIHKENIIASGNNPYLIDMETLSNCQAPLINDKA<br>TMLEHFFYENSQSVFGTMLLPTNSAVSIFDYDIGGISGDDNIETSKWEAFDIKNQGTDNLQFVKESKFITGGCD<br>NIVKLNGEATRARDYYKNIIEGFSDCYKIFIKTPNKVVDILKESEVIIRQVLRPTAVYSKFLEASTYPTYLTNEES<br>FRGLFAKLDNLEEVKEKKKAQIEIDSLYEFDIPYFYSDLNTTNIYSVKGKVDNYINYSVMDAISGKARKFSEKDL<br>KVQIYYINLALSTQPNTSEMIYKYNLFSKSQNLKNITINHIAKQIGDVIEEKLIWDYRKQSCFFMIDTVVKEKRK<br>YCSMDSSLYGSGGVILYYLSLYKATCDLKYKHIVEGLINGHIGLHSKKSLEEGIGLFSGLTSLAYIYYQCYRVLGKK<br>QYLEDVLEVLKEIEENLHMWDELDLSTGLSGVIIFLSELYKTENNEQYLLIANGLADKLYKLVLKANFNMLTGL<br>AHGYAGVAWALYSIGHIVNNEDYINCAMKCIEEENMYYDSEKNNWKDKRNGSSESFYWCYGAFGIGIARLKM<br>YEIAHNDILLKDIEKCRYIYINYNLINSKYNHSLCHGLTGNLSAIKMFSDFYKNDNKLKKKYKEMLDILLNDVV<br>NGKVIWGDKELIEDYSFMIGLSGIGYELLRHEATNSVNILALEV

>cloA1
ATGCAAAATTATGAAAGCAAAGCAGGATTTATTTCTGAAATGGAACTAGATGAATTAGTTTCAAACAAAAC
TGTTGGTGGTGCTACTACTGTACCTTGTGCAATTGCTATTATTGGAATTACACTTTCAGCTGGTATCTGCCC
AACTTCAGCATGCTCAAAAGATTGTCCATGGAATAACTAA

> CloA1 precursor peptide
MQNYESKAGFISEMELDELVSNKTVGGATTVPCAIAIIGITLSAGICPTSACSKDCPWNN

> ***cloPt2***
ATGATCAAGAGAAGCGGTATACGAATAATTAAACAATTAACAGAAACAGAATGTGGCTTATGTTGTTGTGC
CATGATATTGAGATATTACGGAAGTAAGGAATCAATAAGAGAGTTACAAGATTATATGGATGTCGGCAGAG
ATGGGATATCTATGTTTCAGATTAAGCATTTCTTAAATGAAAAAGGTGTTTCTGCCAAAGTATATGAAGTC
AATGAGATTGATAAGTTAGTTCATATAGATAAACCATTTATATGTTATTGGAATCAAAAGCATTTCGTTAT
TGTAGAAAAAATAAAAAATAATATGTTTTATATAGCAGATCCAGCAGATGGGAAAGTAGTTCTTAATAGAG
AAGAATTTTCTAAAAAATTTTCAAAAACCATTTTAGTAAGTGATGTTACGGAAGCATTTAAACCTACGAAA
AATAGAAATTACAATCCGTGGATAACTATTTTACAGTATCTTAAAGAAAATAAAATATTAATTCTTGAGAT
TTTGATATTGCTTGGAATAACTTATGGAATAACGTTGGAAATTCCTAATATTGTACAAAAAATAATTGATA
GGACAGGGACGGAAACTAATTCATCATTCTTGAATATTTTTCTTTTGATGCTAGGAGCATGTATAATTGCAT
TTTTCGTGAGTTACTTATTTAAAGGAATAAAAATAATAGCCTTAAATGTGTTTTTAGGAAGAAAACTTGAA
GCAAATACGTATAGACATCTCTTACAACTTCCATATAAGTTTTTTGAAACTAGATCAACTGGTGACTTGTTA
TATCGTATTCAAGGTACCACCAGTATTAAACAGATGTTGTCTACTCAGATTGTTGGAGGCGTAATTGATATT
GGATCTGTTATTGCAATAATTTTTTATATGCAACAGAAGTCAGTGTTGCTTACAATTTGTGCTTTTGCCTTG
TTCATGATAAATATTATAGTGATTTTAGTTATTCAACCTAAACTTACACAAGCTATTAATGGAGAGATTGTT
GAACAGTCCAAATCTCAAACAGCTCAAATAGAATCATTATATTCAATTATTTCAATAAAGATTTCAGCTATG
GAAGATTTGATCTATAAGAATTGGAGCAACATTTATGAGAGTGTTGTTGAAATGTTTCAAAAGAGGATGCT
AATTTCAAATGCATATAGTGCTATTATGGCTGTACTTCAAAACTTTTCACCGATTTTAATATTGTGTTTAGG
AATTAATGAGTATTATAAGGGAAACATTACCATAGGCGAAGTAATTGCGTTTCAAGCTATTTCTTCAACATT
TTTTAATTTGGGAATGTCAATAGTAAATGTATACCCTCAATTTATTTCTGCATCACAATATTTAGATAGGAT
AGCAGATATTTGGAACAGGGAAGCTGAATTAGAGGATGAAAATGCAATTGTTAGAGAAATAAATGGTGATA
TAGAGTTAGAGGATGTATGTTTTTCATATTCAAAAAACTCAAAAAATGTACTTGAAAATATTTCAATGAAT
ATTAAAAGAGGAACTAAAGTTGCAATTGTGGGGGCTTCTGGATCAGGAAAAAGTTCACTAAGTAAAATTTT
AGTTGGGTTGTACAAGCCTACAAAAGGGACAATAAGATTTGATGGAATTCCTATTGAAAAATATGATAGGA
AAGCTATCTGTAGGCAAATGGGAATCGTACCTCAGGATGCTATGTTATTTAATAAAAGTATATATGAAAAT
ATAGTAATGGGAAATTTCAATATAACATTAGAACAGGTAGAAGAAATTACAAAGATTGCATGCATACATGA
TGAAATCAAAGCAATGCCCATGGGATACCATACAATAATATCTGAAATGGGTATGAATTTATCAGGAGGAC
AGAGACAGAGAATTTTATTAGCAAGATCAATGCTCTCGAATCCAAAGATTTTGGTGTTAGATGAAGCAACT
AGTAGCTTAGATAATATTAATGAGAGAAAAATATCAAATTATTTATCTGGTATTGGATGCACAAGAATTAT
TATTGCGCATAGGTTGTCTACTATAGTTGATGCAGATGTTATATTCATAATGAAAAATGGACAAATTGCAG
AATATGGTAAGCATGAAGAGTTGATAAGTAAGAATGGTGAATATAAAAAATTATATTATATCGGTAGAGAT
ATAGATAATTTGAATGTTGTATAA

> CloPt2
MIKRSGIRIIKQLTETECGLCCCAMILRYYGSKESIRELQDYMDVGRDGISMFQIKHFLNEKGVSAKVYEVNEID
KLVHIDKPFICYWNQKHFVIVEKIKNNMFYIADPADGKVVLNREEFSKKFSKTILVSDVTEAFKPTKNRNYNP
WITILQYLKENKILILEILILLGITYGITLEIPNIVQKIIDRTGTETNSSFLNIFLLMLGACIIAFFVSYLFKGIKIIALN
VFLGRKLEANTYRHLLQLPYKFFETRSTGDLLYRIQGTTSIKQMLSTQIVGGVIDIGSVIAIIFYMQQKSVLLTIC
AFALFMINIIVILVIQPKLTQAINGEIVEQSKSQTAQIESLYSIISIKISAMEDLIYKNWSNIYESVVEMFQKRMLIS
NAYSAIMAVLQNFSPILILCLGINEYYKGNITIGEVIAFQAISSTFFNLGMSIVNVYPQFISASQYLDRIADIWNRE
AELEDENAIVREINGDIELEDVCFSYSKNSKNVLENISMNIKRGTKVAIVGASGSGKSSLSKILVGLYKPTKGTIR
FDGIPIEKYDRKAICRQMGIVPQDAMLFNKSIYENIVMGNFNITLEQVEEITKIACIHDEIKAMPMGYHTIISEM
GMNLSGGQRQRILLARSMLSNPKILVLDEATSSLDNINERKISNYLSGIGCTRIIIAHRLSTIVDADVIFIMKNGQI
AEYGKHEELISKNGEYKKLYYIGRDIDNLNVV

> ***cloM2***
ATGGAAGATATGATAAGTTATTACGAAAAATGCTGGATGAAATTATATCCAGAAATGAAGAGTATAGAAGA
GTTAAATACATATCTGAAAAAAGTATTTGGAAAGAGTCTTAAAGAAAAGTTGCAAAATGAAAGAGTAGAAA

AAGTATCCGTAAAAGAGTACAACAGAAATTTAAAAATGTTTTTAGATGACAACATGCTAGAGAACATGAGA
TTTAGTAGGTTTTATGGACCAATTATGGTCGAATATATAGAGAATTTACCTAAATATATTGAAAAAACGAT
GATAGTGAAAAATATTAAACTGTTTATGGAAAGTATGATTCTTCAGCTATCTGACTTAATGTGTAGTATAG
CTTTTCGTACAATGGTTTTTGAAATAAATAATGCGAAAAATAAAAATCTACTTAAAGGAGAGAGTCCTGAA
GAGAGATATAAATATTTTAATAATGAATTGTTGGATGACTATCAATACCGAAAAAGTTTATATAGTGAGTA
TTGTTTTCTAGTTGAAACTTTAGATGAGTGTGCCAAAAATTTTGTGAAATACATTGAAGAAATACTTGTAA
ACACAAGTAAAAATATGTGTAGAATTCAAAGTGACGTTAATTCAAATATTGAATTAGGTAAACTCATAAAT
ATTGAATTTGCTTTGGGAGATACTCATTGTAGAGGAAAAAGTGTAGCAAAACTGATTTTTGAAAATACAAT
AATTTATTATAAGCCTAGAAATAGTATAATTGATAACAAATTCCAATCAGTGCTTAATTTAATAAACGAAA
AAGGTATACTTTCTGGTAGGAAATACAGAGTTATGAATATTCATGGAACAAGTGAATGTGGTTGGTTTGAA
AATATTAAGTATGAAGAATGTAGAAGTATAGATAATGTTCATGATTATTATTTAAAGATAGGCGGCTTAAT
TGGCATTTTGTATTTCTTTAATGCAACAGATTTTCATCATGAAAATATTATAGCTTGTGCTGAAAATCCTAT
GTTAATAGATTTAGAATCAATATTTAGTGTAGAGATGAAAAGTAAAGTTTTTGATGAAAATAGTGCTTATA
ATAATGCAATTGAATATTTAAAATCAAGTGTACAATCGATTGGAATATTACCTAATAAGTTACATATTGGT
GATTTAGATGACAAGTATGAAACAGGAGGTATTGTTTATAAAGAAAAACAAGTTGCACCAATAAAATCTTT
AAAAGTTGTTAATGATGCATCTGATGGTATCCGTACGGAACTAGTAAATTCTATTATTGAAGGAAATCTTA
ATGCACCTAAATATAATGGCAATATTATAAACCCTAAAGAGTATGTTGAAGATATTAAGGAGGGATTTAGG
TTAGTTTATAAATGGATTCTAGGCAACAAGAAAGAATTTATTGAATTTGTTGAAACGTCATTTAGTGAAAC
AAAAATAAGGATTATATTGAAGCCCACATTTATGTATGCACAAATAAATTCAATAGCAAAACATCCTAATT
TTATGAGTAGCGAAGATGAGAATGAACTTATAAATGCAAGAATAGGTATATACGCAGATAATATCGATATT
ATTAAATCAGAAATAAGATCTTTAAAAAGATATGAGATACCTTATTTTTCAGCGTTATTTAACGAAGAAAA
ATTATTTGATGAAGATGAAAATGTATTAGAATCAAGATTAATTATTAGTCCCCAACTTTTATTTAGAAACA
AAGTATGTAAAGCTACTGAAATAGATTTGAATAATCAAATAGATTTTATAAGTATTTCATTTTTAAGTAAA
AATCCAGAAGAGCTTAGAACTGGAATTCATTATGTTGAAGATGCAGTTGAAATCATCAATACTGACAGTTA
TCTAAATGTTGCAAAAGAAATTGGTGATTATTTATATAGCATAGCAATTATCGGTGAAAATCAGCATGGCA
AAAGTGATGCAACATGGATTGGAAGTGCTGTTTCAAAAATAGATGTTAATGATTGGACTTATAGTGTTTCA
GATTTAGATTTATATAATGGAAATAGTGGTATAGCATTATTTCTATTAAATTTATGGAAAGTTACAAAGGA
TAAAAAATATCTTGATTTAGCAATACAGGCAGCAGAACTTATTATAAGCATAATTAAAAATAAGACATTTA
ATCATTCTACTCTTATAGGAGGGTTTAATGGTATCGGCTCTTATATTTACATAATTAGTAAATTAGTGGTGA
ATACCAATGATGAATATTTTTATAGTACGTTAATAGAATCTATTGACTTATTAGAAGAAAGAATTGAAGCC
GCTTCAGAAATGGATTTAGTGGCTGGAGCAAGTGGAATGCTTGCAGTTTTATTGAATGTATATAGTGAAAT
TGATGATAAATTAATTAAAGAAAAAGTAAAACCACTTCTATATATGTTATTTTACAAGATCCAAGAGAATG
TCAAAAGCGGTGGGAAACTAATTAGATATTCTGGTTTTGGACATGGAATTGCAGGGTGTATTCCATACTTGT
ACAAGTTGTATTTAATAGATGAAAATAGAGAAGTCTATCAGTTATTTAGTGAGTTATTGTCATATGAACGC
GATCATTTCTATAGTAAGGAAGAAAAGGATTGGGTTATGTCTGATGATGAGGTCAATTATTCAAAAGCATG
GTGTCATGGTGCACCAGGAATATTACTCGAAAAGATTATTCTAAAGGAATTAGGTTACGAGGATGAGTATT
TGGATCAAGAGATCAAAGTGGCTTTAAATAATATTAAGAAAAAATGTATTGGTAATAATATAGTGTATTGT
CATGGAGATATAGGAAACTTAGATATTATACAATATGCAGCAAAGATCAGCAAAGATGAAAAAATGATTAA
GGAATGTAGTAACACATATGATAAGTTATTTCAACTACATATAAAAAATAACTGGAATAGTGAAGCGTCTG
CATATAGTAAGTGTAAAGGATAATGGTTGGAGTATCTGGTATTGGGCTATCATTACTGAGGATGATAAAT
AAGTATGATATAGATGATTTTCTTTGGCTAAGTTAA

> CloM2

MEDMISYYEKCWMKLYPEMKSIEELNTYLKKVFGKSLKEKLQNERVEKVSVKEYNRNLKMFLDDNMLENM
RFSRFYGPIMVEYIENLPKYIEKTMIVKNIKLFMESMILQLSDLMCSIAFRTMVFEINNAKNKNLLKGESPEERY
KYFNNELLDDYQYRKSLYSEYCFLVETLDECAKNFVKYIEEILVNTSKNMCRIQSDVNSNIELGKLINIEFALGD
THCRGKSVAKLIFENTIIYYKPRNSIIDNKFQSVLNLINEKGILSGRKYRVMNIHGTSECGWFENIKYEECRSIDN
VHDYYLKIGGLIGILYFFNATDFHHENIIACAENPMLIDLESIFSVEMKSKVFDENSAYNNAIEYLKSSVQSIGILP
NKLHIGDLDDKYETGGIVYKEKQVAPIKSLKVVNDASDGIRTELVNSIIEGNLNAPKYNGNIINPKEYVEDIKEG
FRLVYKWILGNKKEFIEFVETSFSETKIRIILKPTFMYAQINSIAKHPNFMSSEDENELINARIGIYADNIDIIKSEI
RSLKRYEIPYFSALFNEEKLFDEDENVLESRLIISPQLLFRNKVCKATEIDLNNQIDFISISFLSKNPEELRTGIHY
VEDAVEIINTDSYLNVAKEIGDYLYSIAIIGENQHGKSDATWIGSAVSKIDVNDWTYSVSDLDLYNGNSGIALFL
LNLWKVTKDKKYLDLAIQAAELIISIIKNKTFNHSTLIGGFNGIGSYIYIISKLVVNTNDEYFYSTLIESIDLLEERI

EAASEMDLVAGASGMLAVLLNVYSEIDDKLIKEKVKPLLYMLFYKIQENVKSGGKLIRYSGFGHGIAGCIPYLYKLYLIDENREVYQLFSELLSYERDHFYSKEEKDWVMSDDEVNYSKAWCHGAPGILLEKIILKELGYEDEYLDQEIKVALNNIKKKCIGNNIVYCHGDIGNLDIIQYAAKISKDEKMIKECSNTYDKLFQLHIKNNWNSEASAYSKCKGIMVGVSGIGLSLLRMINKYDIDDFLWLS
>***cloA2***<br>ATGAAGAATTATGAAGAATTATTTAATGAAGTTAATGAAAATGCTTCATTACAAGCAGAATTAAACGGTGGTAGTATTGCAACTACTATAGTTTGCACAATTGCACAATCTCTTTTAGGTTGTGTTGGTAGCTATGTTCTTGGAAACAAGGGATATGGTTGTACAGTTACAAATGAATGTATGAGTAACTGTAGATAA
**> CloA2 precursor peptide**<br>MKNYEELFNEVNENASLQAELNGGSIATTIVCTIAQSLLGCVGSYVLGNKGYGCTVTNECMSNCR

Table S2. Precursor peptides within the clostridial clade, selected based on the criteria: the BGCs contained all the essential genes (the precursor and the biosynthetic enzyme), the precursors contained residues that could be recognized by the peptidase (GG or GA), and formed heterocycles specific for lanthipeptides (the amino acid sequence contained serine, threonine, and cysteine), and they all belonged to the same transcriptional unit. The table includes molecular weight (Mw), isoelectric point (IP), and grand average of hydropathicity (Gravy) values. These values were computed using the ProtParam tool for the core peptide, excluding any post-translational modifications. Additionally, in pink the characteristic cleavage sites of the C39 peptidase, presence of serine (S), threonine (T), and cysteine (C), and the predicted cycles by RiPPMiner.

Bacteria	BGC Internal Code	Lanthipeptides	MW (Da)	IP	GRAVY	S+T	C	Cycles
Blautia wexlerae DSM 19850	AXVN01000112	MKKNYRNPMTRPENFMNPAGNVMKEIKEADLNNFSAGAGEPRVSDGSQFCTSTKECNWGTIMFVCC	3086.49	4.68	-0.132	6	4	3
Caldicellulosiruptor bescii DSM 6725	NC_012034	MKESTIIKNPVLRNKVNAKIYNPAGDIVKEIQEQNLPEQAGGGTPTVVVGVISAVTAVTNLAFSIDQAITKYYACSLVYTYSAECRSDGRSCRMR	5696.53	8.61	0.323	12	3	2
Caldicellulosiruptor kristjanssonii 177R1B	NC_014721	MVETYKKNLYGITFEELEEGEMQELVGGGPEITTVIESIKAATAFSTLTCVGSAAASLIIATVITYIVTK	4226.98	6.13	1.107	12	1	1
		MNSTIESRIGICFEQLNEEEMLEAMGGNPWLGTPTTAALSTTVACGVVTGLVSAAVSVTVVITKKL	3884.59	9.31	0.995	11	1	1
Caldicellulosiruptor lactoaceticus 6A	NZ_AEKD0100002	MNSTIESRIGICFEQLNEEEMLEAMGGNPWLGTPTTAALSTTFVCGVVSGLVSAAVSVTVVITKKL	3946.66	9.31	1.018	11	1	1
Caldicellulosiruptor sp. Rt8.B8	LACO01000001-R3	MVETYKKNLYGITFEELEEGEMQELVGGGPEITTVIESIKAATAFSTLTCVGSAAASLIIATVITYIVTK	4226.98	6.13	1.107	12	1	1
		MNSTIESRIGICFEQLNEEEMLEAMGGNPWLGTPTTAALSTTVACGVVTGLVSAAVSVTVVITKKL	3884.59	9.31	0.995	11	1	1

	LACO01000001-R5	MKESTIIKNPVLRNKVNAKIYNPAGDIVKEIQEQNLPEQAGGGTPTVVVGVISAVTAVTNLAFSIDQAITKYYACSLVYTYSAECRSDGRSCRMR	5696.53	8.61	0.323	12	3	2
Clostridium cellulovorans 743B	NC_014393 -R6	MANYKIGAIFEQKNYEEMASSQMTGGDGFVTVTSPQYTLSCCITWTIPSIKLTV	3131.65	5.82	0.614	9	2	2
Clostridium cellulovorans 743B	NC_014393 -R18	MQNYESKAGFISEMELDELVSNKTVGGATTVPCAIAIIGITLSAGICPTSACSKDCPWNN	3349.90	5.81	0.676	7	4	4
		MKNYEELFNEVNENASLQAELNGGSIATTIVCTIAQSLLGCVGSYVLGNKGYGCTVTNECMSNCR	4288.97	7.82	0.526	9	5	5
Clostridium scindens ATCC 35704	NZ_DS499711 - R1	LDIPAMDLFMGMLILGGCHSSATPFLSASSECLATFSNLEMQHSPIPVCNFR	3870.36	6.00	0.075	9	3	3
Clostridium sp. BL8	AUPA01000006 - R1	MADYQKVTGFVSVQELEEVTEVDNGAIAWVSVLATAAFTVKLASAVVCETGACTGYCN	3291.84	6.02	1.197	6	3	3
		MKKYNDITGFVSVEELEEVSNEAQGGIALSAITFVTGTVIWIATRAVCETGACTSYCK	3408.01	7.95	0.988	8	3	3
		MQNNYNLSTGFVSIEELEEASNDIGVAGAFTTIACAAIGLSIAILTVAACPTESGACTGYCR	3320.90	6.01	1.171	7	4	4
		MKNIEMLKNPVLRTKYSVNEINPAGDLLTEVTEQDFTISVSGGYDSAKLGNQGSDCSWSRECQRICNWISYGSGGWFGC	4079.44	6.09	-0.619	6	4	3
Clostridium sp. KNHs209	JPNB01000003 - R2	MRNDILNLTNPMEEKELEQILGGGNGVIKTISHECAMNTWQFLFTCCS	2791.22	6.72	0.292	5	3	3
Dorea sp. 5-2	ASTD01000045 - R1	MQQNSNLDYAGDLSVELGEIEKLIPKEEQVEGASTSTLMCGTYFTLICC	1815.16	5.54	1.176	6	3	3
Eubacterium plexicaudatum ASF492	AQFT01000101 - R1	MRDENKKTNEVSGEAFEDLTISEMAEVQGAGDMEGELTTPVCVVIATASASVGLAKTFKGKC	3255.81	6.21	0.539	6	2	2
Lachnospiraceae bacterium 2_1_58FAA	ACTO01000067 - R1	MIDASILLYPIYGGVLMRSKRIPAEEQYRLIMECRQSGLTDHQWCVEHDIKPGTFYNWVKRLRQKGCVDLPASTG	7233.35	8.99	-0.640	6	3	2
Lachnospiraceae bacterium 3_1_57FAA_CT1	ACTP01000039 - R1	LSGTKNRAGASKRRLTLCIRILYEMNGEKISGCSS	2929.46	9.39	-0.162	5	2	2

Roseburia faecis	CYXV01000003 - R1	MEIKSILIKDTTREERIRIVQEGLNQCGGACDFCNGCDNLGGGSVDAFYEPYINGEKELREINEEYRSNSGLVK	5034.45	4.25	-0.674	3	3	1
		MKDLRNPLTRTENFEHPSGNIMKELTEAELNSVAAGAGVARNSGGIACTLTGECNIGTHIKFCCYD	3075.50	6.75	0.263	4	4	3

Table S3. RMSD values resulting from the structural alignments between PCAT1 and the CloA1 and CloA2 mature peptides proteins models predicted by AlphaFold.

PCAT1	CloA1 pLDDT=83.9	CloA2 pLDDT=84.8
Protein domains	RMSD (Å)	RMSD (Å)
Complete protein	3.211	2.924
C39 peptidase	0.982	0.86
ABC membrane	3.25	2.962
ABC transport	1.159	1.204

Table S4. RMSD values resulting from the structural alignments between CylM and the CloM1 and CloM2 protein models predicted by AlphaFold.

CylM	CloM1 pLDDT=87.9	CloM2 pLDDT=88.8
Domain	RMSD (Å)	RMSD (Å)
Complete protein	4.652	6.261
Dehydratase	1.607	1.507
N-Lobe	1.186	1.244
C-Lobe	1.733	2.145
KA	0.568	0.901
Ka11	0.96	0.602
Cyclase	4.888	3.532*

Table S5. Resistance profile of all the strains used in antimicrobial assays.

Antibiotic/ Bacteria	*S. aureus* ATCC 43300	*S. epidermidis* MIQ43	*P. aeruginosa* ATCC PA14	*P. aeruginosa* MIQPA25	*E. coli* K12 IM08B	*A. baumannii* 747
Cephalothin	R	-	R	-	-	-
Cefazolin	R	-	R	-	-	-
Cefotaxime	R	-	S	R	-	S
Aztreonam	R	-	S	R	-	-
Penicillin G	R	R	R	-	-	I
Oxacillin	R	R	R	-	-	-
Ampicillin	M	R	R	-	-	R
Ticarcillin	M	-	S	-	-	-

Mezlocillin	M	-	S	-	-	-
Imipenem	S	-	-	S	-	S
Clindamycin	-	R	-	-	-	-
Erythromycin	R	R	-	-	-	-
TMT-SFM	R	R	-	-	-	-
Rifampicin	-	S	-	-	-	-
Vancomycin	S	S	-	-	-	S
Linezolid	-	S	-	-	-	-
Minocycline	-	S	-	-	-	-
Carbenicillin	-	-	-	R	-	-
Piperacillin	-	-	-	R	-	I
Ceftriaxone	-	-	-	R	-	-
Ceftazidime	-	-	-	M	-	R
Levofloxacin	-	-	-	M	-	S
Ofloxacin	-	-	-	M	-	S
Meropenem	-	-	-	S	-	S
Amikacin	-	-	-	S	-	S
Gentamicin	-	-	-	S	-	-
Ciprofloxacin	-	-	-	S	-	S
Norfloxacin	-	-	-	S	-	-
Colistin	-	-	-	S	-	S
Polymyxin	-	-	-	S	-	-
Streptomycin	-	-	-	-	R	-

Table S6. Ions detected from the MS spectrum of cellulosin harboring 6 net dehydrations.

**Amino acid Sequence** GSSHHHHHHSQDPNSSSARLQKNYEELFNEVNENASLQAELNGGSIATTIVCTIAQSLLGCVGSYVLGNKGYGCTVTNECMSNCRC $C_{383}H_{601}N_{119}O_{133}S_7$	**Average mass**	9225.0425	**Isotopic Mass**	9219.1968
**Ions**	**Expected mass**	**Measured mass**	**Error (ppm)**	**Charge**
**M-6H2O**	9116.9508	-	-	
**$[M-6H_2O+7H]^{7+}$**	1303.4215	1303.42834	6.84	+7

**$[M\text{-}6H_2O+8H]^{9+}$**	1140.6189	1140.62655	7.65	+8
**$[M\text{-}6H_2O+9H]^{9+}$**	1013.9945	1014.00425	9.75	+9
**$[M\text{-}6H_2O+10H]^{10+}$**	912.6951	912.70222	7.12	+10
**$[M\text{-}6H_2O+11H]^{11+}$**	829.8137	829.8293	15.6	+11
**$[M\text{-}5H_2O+11H]^{8+}$**	1142.8689	1142.87899	10.09	+8

Table S7. Ions detected from the MS spectrum of the cellulosin tryptic fragment**.**

**Amino acid Sequence**<br><br>NYEELFNEVNENASLQ<br>AELNGGSIATTIVCTIAQ<br>SLLGCVGSYVLGNKGY<br>GCTVTNECMSNCRC<br>$C_{283}H_{452}N_{78}O_{100}S_7$	**Average mass**	6771.5345	**Isotopic Mass**	6767.0726
**Ions**	**Expected mass**	**Measured mass**	**Error (ppm)**	**Charge**
**M-6H2O**	6663.4429			
**$[M\text{-}6H_2O+3H]^{3+}$**	2222.1476	2222.1512	3.6	+3
**$[M\text{-}6H_2O+4H]^{4+}$**	1666.8607	1666.85197	-8.73	+4
**$[M\text{-}6H_2O+5H]^{5+}$**	1333.6886	1333.68256	-6.04	+5
**$[M\text{-}5H_2O+5H]^{5+}$**	1337.2886	1337.30433	15.73	+5

Table S8. Ions detected from the MS spectrum of cellulosin after an iodoacetamide alkylation tryptic fragment.

**Amino acid Sequence**<br><br>NYEELFNEVNENASLQ AELNGGSIATTIVCTIAQ SLLGCVGSYVLGNKGY GCTVTNECMSNCRC $C_{283}H_{452}N_{78}O_{100}S_7$	**Average mass**	6771.5345	**Isotopic Mass**	6767.0726
**Ions**	**Expected mass**	**Measured mass**	**Error (ppm)**	**Charge**
**[M-6H2O+IAA+5H]$^{5+}$**	6720.4429			
**[M-6H_2O+IAA+3H]$^{3+}$**	2241.1476	2241.15814	10.54	+3
**[M-6H_2O+IAA+4H]$^{4+}$**	1681.1107	1681.12413	13.43	+4
**[M-6H_2O+IAA+5H]$^{5+}$**	1345.0886	1345.09924	10.64	+5

Table S9. Ions detected from the MS spectrum of cellulosin without any dehydration.

**Amino acid Sequence**<br><br>GSSHHHHHHSQDPNSSS ARLQQNYESKAGFISEM ELDELVSNKTVGGATT VPCAIAIIGITLSAGICPT SACSKDCPWNNC $C_{360}H_{564}N_{108}O_{122}S_6$	**Average mass**	8549.3705	**Isotopic Mass**	8543.9573
**Ions**	**Expected mass**	**Measured mass**	**Error (ppm)**	**Charge**
**M**	8543.9573			
**[M+6H]$^{6+}$**	1425.8951	1425.8951	-5.431	+6

**$[M+7H]^{7+}$**	1222.3386	1222.3386	-4.816	+7
**$[M+8H]^{9+}$**	1069.6713	1069.6713	-4.063	+8
**$[M+9H]^{9+}$**	950.9301	950.9301	-3.792	+9
**$[M+10H]^{10+}$**	855.9371	855.9371	-3.02	+10

Table S10. Fragment ions detected from the MS/MS of the tryptic fragment from N24 to C-terminal of the clostrisin harboring 6 net dehydrations.

Ions	Expected mass	Measured mass	Error (ppm)	Charge
b12	462.1928	461.9928	0.2	3
b19	1042.4505	1046.2505	-3.8	2
b31	866.4009	866.8009	-0.4	4
b38	1432.6359	1432.4359	0.2	3
b40	902.415	891.215	11.2	5
b40	1127.7669	1132.5669	-4.8	4
b41	1149.5249	1143.4249	6.1	4
b42	1178.0356	1161.2356	16.8	4
b45	1260.0884	1276.9884	-16.9	4
b47	1031.0808	1035.6808	-4.6	5
b48	1045.2882	1051.4882	-6.2	5
b48	1306.3584	1293.6584	12.7	4
b49	1065.4977	1065.9977	-0.5	5
b55	985.4658	983.1658	2.3	6
y14	763.2971	769.8971	-6.6	2
y14	1525.5868	1525.0868	0.5	1
y15	814.8017	813.9017	0.9	2
y15	1628.596	1641.596	-13	1
y17	899.8544	902.8544	-3	2
y17	1798.7016	1796.6016	2.1	1
y18	935.373	936.573	-1.2	2
y19	978.889	972.689	6.2	2
y20	1035.431	1039.931	-4.5	2
y23	1171.0076	1174.8076	-3.8	2
y3	350.1129	357.5129	-7.4	1
y30	1511.7018	1525.5018	-13.8	2
y5	633.245	634.245	-1	1
y8	979.376	965.276	14.1	1

Table S11. Fragment ions detected from the MS/MS spectrum of the tryptic fragment from N24 to C-terminal of the clostrisin harboring 6 net dehydrations and 1 alkylation.

Ions	Expected mass	Measured mass	Error (ppm)	Charge	Modification(s)
b_2	278.1135	278.1212	7.7	+1	
b_3	407.1561	407.1621	6.0	+1	
b_4	536.1987	536.2047	6.0	+1	
b_5	649.2828	649.2868	4.0	+1	
b_6	796.3512	796.3528	1.6	+1	
b_8	1039.4367	1039.4527	16.0	+1	
y_{38}	1955.3676	1955.3575	-10.1	+2	6 dehydrations 1 alkylation (6DH 1AK)
y_{37}	1913.849	1913.8394	-9.6	+2	5DH 1AK
y_{36}	1857.307	1857.3115	4.5	+2	5DH 1AK
y_{35}	1807.7728	1807.7772	4.4	+2	5DH 1AK
y_{24}	1281.5155	1281.5392	23.7	+2	3DH 1AK

Table S12. Fragment ions detected from the MS/MS spectrum of the tryptic fragment from N24 to C-terminal of the clostrisin harboring 5 net dehydrations.

Ions	Expected mass	Measured mass	Error (ppm)	Charge	Modification(s)
b_2	278.1135	278.112	-1.5	+1	
b_3	407.1561	407.1527	-3.4	+1	
b_4	536.1987	536.195	-3.7	+1	
b_5	649.2828	649.2798	-3.0	+1	
b_6	796.3512	796.3472	-4.0	+1	
b_7	910.3941	910.3971	3.0	+1	
b_8	1039.4367	1039.4385	1.8	+1	
b_9	1138.5051	1138.4998	-5.3	+1	
b_{11}	1381.5907	1381.5893	-1.4	+1	
b_{13}	783.839	783.8359	-3.1	+2	
y_{51}	1704.7927	1704.7775	-15.2	+3	5 dehydrations (5DH)
y_{49}	1638.0874	1638.0852	-2.2	+3	5DH
y_{48}	1595.4012	1595.3851	-16.1	+3	5DH
y_{47}	1571.7221	1571.7076	-14.5	+3	5DH
y_{46}	1528.708	1528.6978	-10.2	+3	5DH
y_{45}	1491.0133	1491.0128	-0.5	+3	5DH
y_{44}	1452.999	1452.9898	-9.2	+3	5DH
y_{40}	2021.9153	2021.9089	-6.4	+2	5DH
y_{39}	1986.3967	1986.3801	-16.6	+2	5DH
y_{38}	1935.8729	1935.8639	-9.0	+2	5DH

y_{37}	1894.3543	1894.3549	0.6	+2	4DH
y_{36}	1837.8123	1837.8093	-3.0	+2	4DH
y_{35}	1788.2781	1788.2778	-0.3	+2	4DH
y_{24}	1262.0207	1262.0083	-12.4	+2	2DH

Table S13. Fragment ions detected from the MS/MS spectrum of clostrisin.

Ions	Expected mass	Measured mass	Error (ppm)	Charge
**b_5**	509.0106	506.2106	-2.8	+1
**b6**	633.3695	643.2695	9.9	+1
**b7**	796.5284	780.3284	-16.2	+1
**b8**	901.7874	917.3874	15.6	+1
**b9**	534.4268	527.7268	-6.7	+2
**b10**	565.1428	571.2428	6.1	+2
**b11**	637.6721	635.2721	-2.4	+2
**b12**	461.9928	462.1928	0.2	+3
**b12**	700.1855	692.7855	-7.4	+2
**b18**	632.6691	643.2691	10.6	+3
**b19**	698.2028	695.3028	-2.9	+3
**b19**	1046.251	1042.451	-3.8	+2
**b23**	1297.473	1284.073	-13.4	+2
**b27**	1040.362	1025.462	-14.9	+3
**b28**	1056.841	1049.141	-7.7	+3
**b30**	854.7299	838.1299	-16.6	+4
**b31**	866.8009	866.4009	-0.4	+4
**b31**	1145.066	1154.866	9.8	+3
**b33**	919.3196	920.4196	1.1	+4
**b38**	1075.429	1074.729	-0.7	+4
**b38**	1432.436	1432.636	0.2	+3
**b40**	891.215	902.415	11.2	+5
**b40**	1132.567	1127.767	-4.8	+4
**b40**	1494.453	1503.353	8.9	+3
**b41**	1143.425	1149.525	6.1	+4
**b42**	1161.236	1178.036	16.8	+4
**b45 -H2O**	1019.17	1004.67	-14.5	+5
**b45**	1276.988	1260.088	-16.9	+4
**b47**	1035.681	1031.081	-4.6	+5
**b48**	1051.488	1045.288	-6.2	+5
**b48**	1293.658	1306.358	12.7	+4
**b49**	1065.998	1065.498	-0.5	+5
**b50 -H2O**	1082.705	1082.105	-0.6	+5
**b51 -H2O**	1087.319	1101.919	14.6	+5
**b53**	1159.033	1145.533	-13.5	+5
**b55 -H2O**	1163.455	1178.755	15.3	+5
**b56**	1181.165	1196.565	15.4	+5
**y10**	1177.117	1169.417	-7.7	+1

**y17**	902.8544	899.8544	-3	+2
**y17**	1796.602	1798.702	2.1	+1
**y14**	769.8971	763.2971	-6.6	+2
**y14**	1525.087	1525.587	0.5	+1
**y5**	634.245	633.245	-1	+1
**y52**	1319.441	1336.641	17.2	+4
**y15**	813.9017	814.8017	0.9	+2
**y15**	1641.596	1628.596	-13	+1
**y23**	1174.808	1171.008	-3.8	+2
**y19**	972.689	978.889	6.2	+2
**y19**	1959.971	1956.771	-3.2	+1
**y2**	253.67	236.07	-17.6	+1
**y58**	1187.573	1196.573	9	+5
**y11**	1237.754	1240.454	2.7	+1
**y18**	936.573	935.373	-1.2	+2
**y8**	965.276	979.376	14.1	+1
**y40**	983.079	988.479	5.4	+4
**y20**	1039.931	1035.431	-4.5	+2
**y12**	1310.186	1327.486	17.3	+1
**y30**	1008.437	1008.137	-0.3	+3
**y30**	1525.502	1511.702	-13.8	+2
**y41**	1344.147	1346.647	2.5	+3
**y3**	357.5129	350.1129	-7.4	+1
